# Transcriptomic and phenotypic analysis of murine embryonic stem cell derived BMP2^+ ^lineage cells: an insight into mesodermal patterning

**DOI:** 10.1186/gb-2007-8-9-r184

**Published:** 2007-09-04

**Authors:** Michael Xavier Doss, Shuhua Chen, Johannes Winkler, Rita Hippler-Altenburg, Margareta Odenthal, Claudia Wickenhauser, Sridevi Balaraman, Herbert Schulz, Oliver Hummel, Norbert Hübner, Nandini Ghosh-Choudhury, Isaia Sotiriadou, Jürgen Hescheler, Agapios Sachinidis

**Affiliations:** 1 Institute of Neurophysiology, University of Cologne, Robert-Koch Str. 39, 50931 Cologne, Germany; 2 Institute of Pathology, University of Cologne, Joseph-Stelzmann-Str. 9, 50931 Cologne, Germany; 3 Max-Delbrueck-Center for Molecular Medicine - MDC, Robert-Rössle Str. 10, 13092 Berlin, Germany; 4 Department of Pathology, The University of Texas Health Science Center at San Antonio, TX 78229, USA

## Abstract

**Background:**

Bone morphogenetic protein (BMP)2 is a late mesodermal marker expressed during vertebrate development and plays a crucial role in early embryonic development. The nature of the BMP2-expressing cells during the early stages of embryonic development, their transcriptome and cell phenotypes developed from these cells have not yet been characterized.

**Results:**

We generated a transgenic BMP2 embryonic stem (ES) cell lineage expressing both puromycin acetyltransferase and enhanced green fluorescent protein (EGFP) driven by the *BMP2 *promoter. Puromycin resistant and EGFP positive BMP2^+ ^cells with a purity of over 93% were isolated. Complete transcriptome analysis of BMP2^+ ^cells in comparison to the undifferentiated ES cells and the control population from seven-day-old embryoid bodies (EBs; intersection of genes differentially expressed between undifferentiated ES cells and BMP2^+ ^EBs as well as differentially expressed between seven-day-old control EBs and BMP2^+ ^EBs by *t*-test, *p *< 0.01, fold change >2) by microarray analysis led to identification of 479 specifically upregulated and 193 downregulated transcripts. Transcription factors, apoptosis promoting factors and other signaling molecules involved in early embryonic development are mainly upregulated in BMP2^+ ^cells. Long-term differentiation of the BMP2^+ ^cells resulted in neural crest stem cells (NCSCs), smooth muscle cells, epithelial-like cells, neuronal-like cells, osteoblasts and monocytes. Interestingly, development of cardiomyocytes from the BMP2^+ ^cells requires secondary EB formation.

**Conclusion:**

This is the first study to identify the complete transcriptome of BMP2^+ ^cells and cell phenotypes from a mesodermal origin, thus offering an insight into the role of BMP2^+ ^cells during embryonic developmental processes *in vivo*.

## Abstract 2

Transcriptome analysis of BMP2^+ ^cells in comparison to the undifferentiated BMP2 ES cells and the control population from 7-day old embryoid bodies led to the identification of 479 specifically upregulated and 193 downregulated transcripts.

## Background

Bone morphogenetic protein (BMP)2 is a member of the transforming growth factor (TGF)-β superfamily and plays a crucial role in early embryonic patterning as shown by gene ablation studies [1,2]. It is normally expressed in lateral plate mesoderm and extraembryonic mesoderm [1,3]. BMP2^+ ^mesodermal cells at this stage comprise a subset of mesoderm, the lateral plate cardiogenic mesoderm [4]. BMP2 expression immediately follows the transient expression of T-Brachyury in the nascent mesoderm. Interestingly, administration of soluble BMP2 to chick embryo explant cultures induces full cardiac differentiation in stage 5-7 anterior medial mesoderm, a tissue that is normally not cardiogenic [5].

Since BMP2 is a cardiogenic factor as well as expressed in the cardiogenic mesoderm, it is highly imperative to investigate the molecular nature and phenotype of the mesodermal cells expressing BMP2 during the early stages of development in the context of cardiomyogenesis. Also, it has been well documented that BMP2 is a potent apoptotic inducer and a potent neurotrophic factor, acting on target cells in a concentration gradient-dependant manner, mostly through its paracrine mode of action [6-8]. Thus, BMP2 plays a pivotal role not only during cardiomyogenesis but also during other early embryonic patterning and lineage specification. To date, the molecular nature and phenotype of the mesodermal cells expressing BMP2 during the early stages of development have not been characterized, leaving a gap in our understanding of their molecular interactions with target cells and, thus, their role during early embryonic patterning and cell lineage commitment. This is due, in part, to the pleiotrophic effects of BMP2 and largely because of the practical difficulty in isolating pure early stage BMP2-expressing cells in sufficient quantities during early embryonic development *in vivo*. Extensive investigations applying the *in vitro *embryonic stem (ES) cell-based developmental model in the past two decades have proven its value for the elucidation of developmental processes during embryonic development, in particular, the mechanisms by which lineage commitment occurs during early embryogenesis [9].

To circumvent the practical difficulties in the isolation of BMP2-expressing cells in sufficient quantities during embryonic development *in vivo*, and to address the molecular nature and behaviour of the BMP2^+ ^mesodermal cells during their differentiation into specific somatic cell lineages, we first established an ES cell-derived transgenic BMP2 cell lineage expressing both puromycin acetyltransferase and enhanced green fluorescent protein (EGFP) under the control of the *BMP2 *promoter. In order to identify all possible signal transduction pathways and biological processes characteristic of the BMP2^+ ^cells, we performed expression studies using Affymetrix microarrays. Our study on the phenotypic identification of the ES cell-derived BMP2 lineage-specific cells shows that the early BMP2^+ ^population contained a heterogeneous population of predominantly NCSCs and their lineages - smooth muscle cells, epithelial like cells, astrocytes and melanocytes. When the early BMP2^+ ^population was further cultured under certain conditions, it contained cardiomyocytes, macrophages and osteoblasts. Interestingly, these are the cell phenotypes that need BMP2 for their phenotypic induction. Our work clearly demonstrates the presence of a multi-lineage progenitor phenotype resembling NCSCs cells in early ES cell-derived BMP2^+ ^cells. Moreover, identification of the key signal transduction pathways induced or repressed in BMP2^+ ^cells explains the observed potential of BMP2 in modulating early embryonic development, in particular the mesodermal patterning.

## Results and discussion

### Isolation of BMP2^+ ^cells from the transgenic BMP2 ES cell lineage

**Figure 1:**
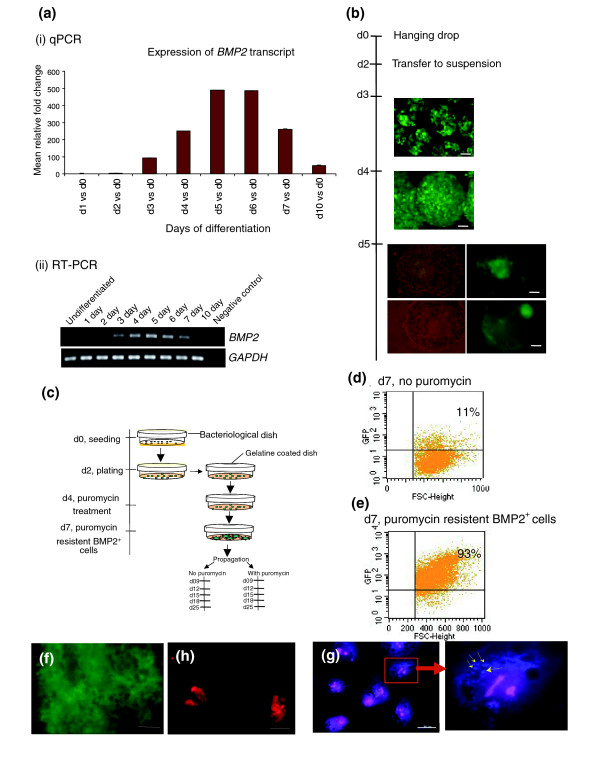
Expression pattern of BMP2 in differentiating EBs. **(a) **Detection of the expression of *BMP2 *by qPCR on the samples from EBs derived from wild-type CGR8 ES cells (i) and RT-PCR (ii) on samples from EBs derived from BMP2 ES cells (for conditions, see Additional data file 14). The qPCR results are presented as the mean of three independent experiments ± standard deviation. **(b) **Expression of EGFP during differentiation of the BMP2 ES cells induced by the conventional hanging drop protocol. Scale bar represents 50 μm. **(c) **Protocol for isolation of puromycin resistant BMP2^+ ^cells after treating the plated four-day-old EBs with 3 μg/ml puromycin for three days. **(d,e) **FACS analysis of the trypsinized untreated control and puromycin resistant BMP2^+ ^cells. **(f) **BMP2^+^, three days after plating in gelatine-coated tissue culture dishes in the presence of 3 μg/ml puromycin. Scale bar represents 50 μm. **(g,h) **Detection of BMP2 in BMP2^+ ^cells (g) or ES cells (h) by immunohistochemistry staining. Stainings were done after the cells were trypsinized and plated on microscopic slides for 24 hours. Scale bar represents 20 μm.

The transgenic BMP2 ES cell lineage was generated with the linearized pBMP2^p^-puro IRES2 EGFP construct by stable transfection. Like its parental wild type CGR8, the BMP2 ES cells do not express BMP2 in the undifferentiated state (Figure 1a(i) and 1a(ii)). Expression of BMP2 during progressive differentiation induced by the hanging drop protocol (see Materials and methods) starts in the three-day-old embryoid bodies (EBs), gradually increases to a maximum in the five-day-old EBs and, thereafter, gradually decreases to a minimum in ten-day-old EBs, in the same manner as that seen in the RT-PCR results (Figure 1a). During the course of differentiation induced by the hanging drop protocol, the EGFP-expressing cells in the three- and four-day-old EBs were found to be scattered (Figure 1b). As differentiation continues, the EGFP fluorescence peaks in the five-day-old EBs and the EGFP-expressing cells are localized to a particular region in every EB, as shown in Figure 1b. The RNAs isolated from these EBs were analyzed for the expression of other candidate markers (*T-bra*, *flk1*, *smooth muscle α-actin*, *neurofilament-H *(*NF-H*) and also *α-fetoprotein *(*AFP*)) to demonstrate that these EBs were differentiating in the normal way as per their parental wild-type EBs (Additional data file 1). Isolation and further characterization of the BMP2^+^, puromycin-resistant cells were optimized according to the protocol described in Figure 1c. Briefly, a single cell suspension of BMP2^+ ^ES cells was seeded in bacteriological dishes for two days to form two-day-old EBs. These were then transferred into gelatine coated tissue culture dishes and cultured for a further two days. Thereafter, plated EBs were treated with 3 μg/ml puromycin for three days. After trypsinization of puromycin-resistant seven-day-old BMP2^+ ^cells, fluorescence-activated cell sorting (FACS) analysis was performed. As demonstrated in Figure 1d,e, after 3 days of puromycin treatment, EGFP fluorescing and puromycin resistant BMP2^+ ^cells (hereafter called BMP2^+ ^cells) accounted for 93% of the cells in the EBs, whereas in the control EBs without puromycin treatment (hereafter called control EBs (seven-day-old EBs)) only 11% of the cells were BMP2^+ ^cells. This result demonstrates a nearly 8.5-fold enrichment of BMP2^+ ^cells in EBs treated with puromycin. As demonstrated in Figure 1f, plating of the BMP2^+ ^cells in gelatine coated tissue culture dishes for another three days in the presence of puromycin results in a bright EGFP-positive BMP2^+ ^cell population. Furthermore, the BMP2 protein was detected by immunostaining using BMP2-specific antibodies. The undifferentiated BMP2 ES cells were included as a negative control (Figure 1h). As demonstrated, BMP2 is detected only in the cytosol, and specifically in vesicles, of the BMP2^+ ^cells (Figure 1g).

### Functional categorization of transcripts upregulated in BMP2^+ ^cells

The Affymetrix data obtained were validated by quantitative real time PCR (qPCR; Additional data file 2). To identify Gene Ontology (GO) [10] categories, Kyoto Encyclopedia of Genes and Genomes (KEGG) pathways [11] and BioCarta pathways [12] specifically enriched in BMP2^+ ^cells, we first analyzed genes that are upregulated in BMP2^+ ^cells in comparison to control EBs. Moreover, to identify BMP2^+ ^cell-specific genes, a three condition comparative analysis of the BMP2^+ ^cells to control EBs and to BMP2 ES cells was made (Tables 1 and 2).

**Table 1:** Functional annotations enriched among genes upregulated* in BMP2^+ ^cells compared to control cells in seven-day-old EBs

Category	Term	Count	*p *value
GOTERM_MF_5	Zinc ion binding	142	3.2E-16
GOTERM_CC_5	Nucleus	279	3.6E-12
GOTERM_BP_5	Transcription	128	2.5E-10
GOTERM_BP_5	Regulation of nucleobase, nucleoside, nucleotide and nucleic acid metabolism	121	5.5E-9
GOTERM_BP_5	Cellular protein metabolism	147	4.9E-8
GOTERM_MF_5	Binding	143	2.9E-7
GOTERM_MF_5	Metal ion binding	61	6.8E-7
GOTERM_MF_5	Transition metal ion binding	74	4.1E-6
GOTERM_MF_5	Cation binding	74	4.6E-6
GOTERM_MF_5	Ion binding	74	4.6E-6
GOTERM_BP_5	Biopolymer modification	83	1.3E-5
GOTERM_BP_5	Response to unfolded protein	12	2.5E-5
GOTERM_MF_5	ATP binding	84	6.8E-5
GOTERM_BP_5	Apoptosis	38	1.2E-4
GOTERM_CC_5	Intracellular membrane-bound organelle	225	2.4E-4
GOTERM_CC_5	Membrane-bound organelle	225	2.4E-4
GOTERM_BP_5	Regulation of protein kinase activity	12	3.1E-4
GOTERM_MF_5	Protein kinase activity	44	4.9E-4
KEGG_PATHWAY	MAPK signaling pathway	24	7.1E-4
GOTERM_BP_5	Nucleobase, nucleoside, nucleotide and nucleic acid metabolism	18	1.7E-3
GOTERM_BP_5	Regulation of programmed cell death	24	1.9E-3
GOTERM_CC_5	Intracellular	244	3.3E-3
GOTERM_BP_5	Response to protein stimulus	6	3.4E-3
GOTERM_CC_5	Vacuole	17	3.4E-3
GOTERM_BP_5	Phosphate metabolism	45	3.7E-3
GOTERM_BP_5	Cell death	16	4.1E-3
GOTERM_BP_5	Death	16	4.1E-3
GOTERM_BP_5	Programmed cell death	16	4.1E-3
GOTERM_BP_5	Negative regulation of cellular metabolism	17	5.6E-3
BIOCARTA	The information-processing pathway at the IFN-β enhancer	4	6.2E-3
GOTERM_BP_5	Regulation of apoptosis	22	6.7E-3
GOTERM_BP_5	Protein kinase cascade	17	6.8E-3
GOTERM_BP_5	Embryonic development	15	7.2E-3
GOTERM_MF_5	Nucleotide binding	41	8.9E-3
GOTERM_CC_5	Intracellular organelle	243	9.0E-3
GOTERM_CC_5	Organelle	243	9.0E-3
GOTERM_BP_5	Negative regulation of progression through cell cycle	10	9.3E-3
GOTERM_BP_5	Regulation of progression through cell cycle	24	9.7E-3
GOTERM_BP_5	Positive regulation of programmed cell death	12	1.2E-2
GOTERM_BP_5	Embryonic limb morphogenesis	7	1.5E-2
GOTERM_MF_5	Pyrophosphatase activity	29	2.1E-2
BIOCARTA	Regulation of transcriptional activity by PML	4	2.2E-2
GOTERM_BP_5	Cellular physiological process	112	2.3E-2
GOTERM_MF_5	Purine nucleotide binding	52	2.4E-2
GOTERM_BP_5	Embryonic development (sensu Mammalia)	7	2.7E-2
GOTERM_CC_5	Lytic vacuole	13	2.8E-2
GOTERM_BP_5	Negative regulation of protein kinase activity	5	2.8E-2
GOTERM_BP_5	Regulation of biological process	34	2.9E-2
GOTERM_CC_5	Cell	256	3.0E-2
GOTERM_BP_5	Regulation of cellular process	29	3.4E-2
GOTERM_BP_5	Regulation of protein biosynthesis	9	3.8E-2
GOTERM_MF_5	Transcription cofactor activity	9	3.8E-2
GOTERM_MF_5	Transcription factor binding	9	4.0E-2
GOTERM_BP_5	Negative regulation of programmed cell death	9	4.2E-2
GOTERM_BP_5	Protein catabolism	13	4.2E-2
GOTERM_BP_5	Regulation of gene expression, epigenetic	3	4.2E-2
GOTERM_MF_5	Protein kinase binding	6	4.3E-2
GOTERM_BP_5	Primary metabolism	35	4.5E-2
GOTERM_BP_5	RNA metabolism	22	4.5E-2
GOTERM_BP_5	Regulation of cellular biosynthesis	9	4.7E-2
GOTERM_MF_5	Guanyl nucleotide binding	14	4.8E-2
GOTERM_BP_5	Reproduction	11	4.8E-2
GOTERM_BP_5	Response to abiotic stimulus	13	4.8E-2
GOTERM_BP_5	Gene silencing	4	5.0E-2
KEGG_PATHWAY	Pantothenate and CoA biosynthesis	4	5.0E-2
GOTERM_BP_5	Physiological process	127	5.1E-2
GOTERM_BP_5	Bone resorption	3	5.2E-2
GOTERM_MF_5	Cysteine-type peptidase activity	9	5.2E-2
GOTERM_MF_5	Ligase activity	17	5.4E-2
GOTERM_BP_5	Response to chemical stimulus	11	5.4E-2
GOTERM_BP_5	Regulation of bone remodeling	4	5.6E-2
GOTERM_BP_5	Biopolymer catabolism	13	6.1E-2
KEGG_PATHWAY	Nitrogen metabolism	4	6.3E-2
GOTERM_BP_5	ER-nuclear signaling pathway	3	6.4E-2
GOTERM_BP_5	Regulation of protein metabolism	13	6.4E-2
GOTERM_CC_5	Nucleolus	12	7.1E-2
GOTERM_BP_5	Protein biosynthesis	30	7.3E-2
GOTERM_MF_5	Transcription corepressor activity	6	7.3E-2
GOTERM_BP_5	Intracellular receptor-mediated signaling pathway	3	7.6E-2
GOTERM_MF_5	Transcription regulator activity	9	7.7E-2
GOTERM_BP_5	Macromolecule biosynthesis	33	8.0E-2
GOTERM_BP_5	Positive regulation of cell proliferation	9	8.1E-2
GOTERM_BP_5	Embryonic hemopoiesis	2	8.2E-2
GOTERM_BP_5	Posttranscriptional gene silencing	2	8.2E-2
GOTERM_BP_5	RNA-mediated gene silencing	2	8.2E-2
GOTERM_BP_5	RNA-mediated posttranscriptional gene silencing	2	8.2E-2
GOTERM_MF_5	Glutaminase activity	2	8.4E-2
GOTERM_MF_5	Ubiquitin-protein ligase activity	16	8.4E-2
GOTERM_BP_5	Eye development	5	8.6E-2
BIOCARTA	Eukaryotic protein translation	3	9.0E-2
GOTERM_BP_5	Amino acid transport	6	9.1E-2
GOTERM_BP_5	Positive regulation of cell activation	5	9.2E-2
GOTERM_BP_5	Development	37	9.3E-2

*Change fold >2, Student's *t*-test *p *value < 0.01. Count indicates the number of genes in the functional annotation category. The *p *value is from gene enrichment in annotation terms calculated by the Fisher's exact *t*-test.

**Table 2:** Functional annotations enriched among genes upregulated* in BMP2^+ ^cells compared to control cells in seven-day-old EBs and undifferentiated BMP2 ES cells

Category	Term	Count	*p *value
GOTERM_MF_5	Zinc ion binding	46	5.6E-5
GOTERM_CC_5	Nucleus	95	3.3E-4
GOTERM_BP_5	Cellular protein metabolism	66	3.3E-4
GOTERM_BP_5	Protein catabolism	10	4.9E-3
GOTERM_BP_5	Apoptosis	17	5.9E-3
GOTERM_BP_5	Biopolymer modification	38	6.0E-3
GOTERM_BP_5	Biopolymer catabolism	10	6.9E-3
GOTERM_BP_5	Positive regulation of programmed cell death	8	9.1E-3
GOTERM_MF_5	ATP binding	30	1.4E-2
GOTERM_BP_5	Response to unfolded protein	5	1.5E-2
GOTERM_BP_5	Transcription	47	1.5E-2
GOTERM_BP_5	Regulation of nucleobase, nucleoside, nucleotide and nucleic acid metabolism	45	2.7E-2
GOTERM_BP_5	Regulation of programmed cell death	11	3.8E-2
GOTERM_BP_5	Regulation of progression through cell cycle	11	5.1E-2
GOTERM_BP_5	Protein kinase cascade	8	5.3E-2
GOTERM_BP_5	Regulation of protein kinase activity	5	5.6E-2
GOTERM_BP_5	Small GTPase mediated signal transduction	9	5.6E-2
GOTERM_BP_5	Post replication repair	2	6.2E-2
GOTERM_MF_5	Ubiquitin-protein ligase activity	8	6.2E-2
GOTERM_BP_5	Regulation of apoptosis	10	7.4E-2
GOTERM_BP_5	Protein transport	16	7.5E-2
GOTERM_MF_5	Protein kinase activity	15	8.3E-2
KEGG_REACTION	Phytoceramide+h2o<=>fattyacid+phytosphingosine	3	8.7E-2
GOTERM_CC_5	Lytic vacuole	6	8.8E-2
KEGG_PATHWAY	MAPK signaling pathway	9	9.5E-2

*Change fold >2, Student's *t*-test *p *value < 0.01. Count indicates the number of genes in the functional annotation category. The *p *value is from gene enrichment in annotation terms calculated by the Fisher's exact *t*-test.

Table 1 indicates different GO terms (level 5) and one KEGG pathway (mitogen-activated protein kinase (MAPK) signaling pathway) that are enriched in the BMP2^+ ^cells compared to control EBs. In the GO categories 'biological process' (GOTERM_BP), 'molecular function' (GOTERM_MF) and 'cellular component' (GOTERM_CC), several relevant categories are pooled, such as transcriptional processes (for zinc ion binding and transcription) and apoptotic processes (for apoptosis, cell death, death, programmed cell death and regulation of apoptosis) (Table 1). Genes for transcriptional activity and associated with apoptosis are also found to be specifically enriched in the BMP2^+ ^cells (Table 2). These results suggest that BMP2 causes direct or indirect induction of apoptotic processes. This hypothesis is supported by the observations that apoptotic effects of BMP2 promote cavitation in EBs and in mouse embryos [13]. The MAPK signaling pathway involved in apoptotic processes [14] seems to be specific for the BMP2^+ ^cell population (Table 2). It is well established that the MAPK signaling pathway is involved in several processes, including cell cycle progression, cellular transformation apoptosis and differentiation (for a review, see [14]). These results suggest that both apoptosis and transcription genes are characteristic gene expression signatures for the BMP2^+ ^cells.

Additional data files 3-5 list all the genes and include the change factor (CF) values belonging to the GO categories 'transcription' and 'apoptosis' and the KEGG 'MAPK pathway', respectively. Among genes highly upregulated specifically in the BMP2^+ ^cells, *Gm397 *(gene model 397) and *Tbx4 *are identified (Additional data file 3). *Tbx4 *has been shown to be expressed in the lateral mesoderm and is involved in limb outgrowth in the mouse [15] whereas the function of *Gm397 *is unknown.

Interestingly, among the MAPKs, several kinases belonging to both the classical and the c-Jun amino-terminal kinase (JNK) and p38 MAP kinase pathway were overexpressed in BMP2^+ ^cells (Additional data file 5, KEGG pathway scheme). The JNK and p38 MAPK pathway is known to be stimulated by serum and stress factors [14]. The most striking gene specifically upregulated in BMP2^+ ^cells was *Hspa1a *(heat shock protein 1A; Additional data file 5), which belongs to the Hsp70 family of stress response genes. Members of this family participate in the process of folding and refolding of misfolded proteins and in the transport of proteins across membranes [16]. *Hsp1a *is also found to be upregulated in chondrons, which includes the chondrocyte and its pericellular matrix, compared to chondrocytes [17].

Table 3 lists the developmental genes that are overexpressed in the BMP2^+ ^cells compared to control EBs. Among these, *Dnmt3l *(DNA (cytosine-5-)-methyltransferase,3-like), *Fgf4 *(fibroblast growth factor 4), *Tdgf1 *(teratocarcinoma derived growth factor), *Zic1 *(zinc finger protein of the cerebellum 1), *Ifrd1 *(interferon-related developmental regulator 1), *Tbx4 *(T-Box 4), and *Neurod1 *(neurogenic differentiation 1) were highly expressed in the BMP2^+ ^cells. DNA methylation of the genome is essential for mammalian development and plays crucial roles in a variety of biological processes including genomic imprinting [18,19]. *Dnmt3l*^*mat*-/- ^mice die before mid-gestation due to an imprinting defect [18]. In addition, Dnmt3L is required for differentiation in the extra-embryonic tissue [18]. Molecular and genetic data indicate that FGF signaling plays a major role in regulating trophoblast proliferation and differentiation [20]. *Fgf4 *is expressed in early embryos, becoming restricted to the inner cell mass (ICM) of the blastocyst and later to the epiblast of the early post-implantation embryo [20].

**Table 3:** Genes of GO category 'development' upregulated at least two-fold* in BMP2^+ ^cells compared to control cells in seven-day-old EBs

Affymetrix ID	Gene name	Fold change
1425035_s_at	*dna *(*cytosine-5-*)*-methyltransferase 3-like*	14.0
1420086_x_at	*fibroblast growth factor 4*	9.4
1450989_at	*teratocarcinoma-derived growth factor*	9.0
1423477_at	*zinc finger protein of the cerebellum 1*	7.5
1416067_at	*interferon-related developmental regulator 1*	7.0
1456033_at	*t-box 4*	6.8
1426412_at	*neurogenic differentiation 1*	5.7
1418640_at	*sir2 alpha*	4.3
1456341_a_at	*kruppel-like factor 9*	4.1
1424607_a_at	*xanthine dehydrogenase*	3.7
1452240_at	*bruno-like 4*, *rna binding protein *(*drosophila*)	3.7
1452179_at	*phd finger protein 17*	3.6
1416455_a_at	*crystallin*, *alpha b*	3.5
1416953_at	*connective tissue growth factor*	3.2
1428334_at	*osteopetrosis associated transmembrane protein 1*	3.1
1418901_at	*ccaat/enhancer binding protein *(*c/ebp*), *beta*	2.8
1421151_a_at	*eph receptor a2*	2.8
1422556_at	*guanine nucleotide binding protein*, *alpha 13*	2.7
1434009_at	*glucocorticoid receptor dna binding factor 1*	2.6
1434054_at	*v-maf musculoaponeurotic fibrosarcoma oncogene family*, *protein g *(*avian*)	2.5
1422057_at	*nodal*	2.5
1436164_at	*solute carrier family 30 *(*zinc transporter*), *member 1*	2.5
1422033_a_at	*ciliary neurotrophic factor*	2.5
1449949_a_at	*coxsackievirus and adenovirus receptor*	2.5
1433455_at	*linker of t-cell receptor pathways*	2.5
1425932_a_at	*cug triplet repeat*, *rna binding protein 1*	2.4
1451383_a_at	*conserved helix-loop-helix ubiquitous kinase*	2.4
1455222_a_at	*upstream binding protein 1*	2.4
1451257_at	*acyl-coa synthetase long-chain family member 6*	2.4
1426858_at	*inhibin beta-b*	2.3
1421624_a_at	*enabled homolog *(*drosophila*)	2.3
1437540_at	*mucolipin 3*	2.3
1429192_at	*sloan-kettering viral oncogene homolog*	2.2
1452438_s_at	*taf4a rna polymerase ii*, *tata box binding protein *(*tbp*)*-associated factor*	2.2
1436907_at	*neuron navigator 1*	2.1
1450986_at	*nucleolar protein 5*	2.0
1416904_at	*muscleblind-like 1 *(*drosophila*)	2.0

*Student's *t*-test, *p *value < 0.01.

Teratocarcinoma-derived growth factor (encoded by *Tdgf1*, also known as *Cripto-1*) plays a pivotal role as a multifunctional modulator during embryogenesis and oncogenesis, and may be involved in stem cell maintenance [21]. NeuroD1 is a member of the basic helix-loop-helix transcription factor family and has been shown to play a major role in development of the nervous system and formation of the endocrine system [22]. The transcription factor ZIC1 plays important roles in patterning the neural plate in early vertebrate development. *Zic1 *expression was detected in the neural plate border, dorsal neural tube, and somites [23]. Moreover, *Zic1 *plays an important role in early patterning of the *Xenopus *presumptive neurectoderm [24].

Interferon-related developmental regulator 1 (IFRD1; also known as PC4, Tis7) is a chromatin-associated protein that induces chromatin condensation and plays multiple roles in cellular processes, including transcription, DNA replication and repair [25]. It is expressed early in the mouse embryo and extra-embryonic tissues during gastrulation and at mid-gestation in restricted structures (such as the central nervous system, kidney, and lung primordia), whereas it is ubiquitously expressed at late gestation [26]. IFRD1 has been shown to act as a coactivator of myogenic differentiation 1 (MyoD1) and myocyte enhancer factor 2C (MEF2C) during myogenesis [27].

The three condition comparative analysis results in a set of seven BMP2^+ ^cell-specific genes (Table 4). Among these, the most prominently regulated genes are *Zic1*, *Ifrd1 *and *Tbx4*, which have been discussed previously. Ciliary neurotrophic factor (CNTF) is of particular interest. CNTF is a cytokine with neurotrophic and differentiating effects on central nervous system cells and myotrophic effects on skeletal muscle [28].

**Table 4:** Genes of GO category 'development' upregulated at least two-fold* in BMP2^+ ^cells compared to control cells in seven-day-old EBs and undifferentiated BMP2 ES cells

Affymetrix ID	Gene name	Fold change BMP2^+ ^versus BMP2 EBs	Fold change BMP2^+ ^versus BMP2 ES cells
1423477_at	*zinc finger protein of the cerebellum 1*	7.5	8.8
1416067_at	*interferon-related developmental regulator 1*	7	7.1
1456033_at	*T-box 4*	6.8	6.4
1434009_at	*RIKEN cDNA 6430596G11 gene*	2.6	3.4
1422033_a_at	*ciliary neurotrophic factor*	2.5	3.2
1425932_a_at	*CUG triplet repeat*, *RNA binding protein 1*	2.4	2.5
1416904_at	*muscleblind-like 1 *(*Drosophila*)	2	2.7

*Student's *t*-test, *p *value < 0.01.

### GO enrichment analysis of the genes downregulated in BMP2^+ ^cells

To identify overrepresented GO categories or KEGG pathways specifically downregulated in BMP2^+ ^cells, we analyzed the data with the DAVID bioinformatics resource [29]. Comparative analysis of the expression level of genes in BMP2^+ ^and in control EBs shows downregulated genes belong to several overrepresented GO categories, such as focal adhesion, TGF-β signaling pathway, extracellular matrix (ECM)-receptor interaction and shh signaling pathway (Table 5). Some overrepresented categories are related to the developmental processes (for example, development, organ development, embryonic development and brain development; (Table 5, entries in bold). This is not surprising, since the seven-day-old control EBs can still develop into various somatic precursor cells, as indicated in the tables (for example, vasculature development and brain development). Notably, GO categories associated with impaired developmental processes appear not to be characteristic of BMP2^+ ^cells when the expression levels of these genes in undifferentiated ES cells are also taken into account. These results clearly show that the BMP2^+ ^cells are more closely related to the undifferentiated ES cells than to the control EBs with regard to their developmental potential and plasticity.

**Table 5:** Functional annotations (GO, KEGG, Biocarta) enriched in transcripts downregulated* in BMP2^+ ^cells compared to control cells in seven-day-old EBs

Category	Term	Count	p value
GOTERM_BP_5	**Development**	82	3.6E-20
GOTERM_BP_5	**Organ development**	57	5.1E-19
GOTERM_BP_5	Morphogenesis	56	1.6E-16
GOTERM_BP_5	Cell differentiation	37	2.1E-11
GOTERM_BP_5	Blood vessel morphogenesis	25	1.7E-10
GOTERM_BP_5	**Embryonic development**	25	1.2E-8
GOTERM_BP_5	**System development**	30	1.9E-8
GOTERM_BP_5	Organ morphogenesis	27	2.3E-8
GOTERM_BP_5	**Vasculature development**	17	2.8E-8
GOTERM_BP_5	**Tube development**	18	5.8E-8
GOTERM_BP_5	Enzyme linked receptor protein signaling pathway	27	6.1E-8
GOTERM_BP_5	Cell migration	28	1.0E-7
GOTERM_BP_5	**Blood vessel development**	16	1.3E-7
GOTERM_BP_5	**Nervous system development**	25	5.5E-7
GOTERM_BP_5	Embryonic limb morphogenesis	12	1.8E-6
GOTERM_BP_5	Angiogenesis	17	3.7E-6
GOTERM_BP_5	Embryonic morphogenesis	14	6.7E-6
GOTERM_BP_5	Cell motility	21	7.3E-6
GOTERM_BP_5	Locomotion	21	8.7E-6
GOTERM_BP_5	Localization of cell	21	8.7E-6
GOTERM_BP_5	Neuron differentiation	24	1.1E-5
GOTERM_BP_5	Steroid biosynthesis	12	1.2E-5
GOTERM_BP_5	**Brain development**	16	1.9E-5
GOTERM_BP_5	**Cell development**	15	2.0E-5
GOTERM_BP_5	Alcohol catabolism	11	3.2E-5
GOTERM_BP_5	Regulation of nucleobase, nucleoside, nucleotide and nucleic acid metabolism	100	3.3E-5
GOTERM_BP_5	**Tissue development**	12	6.8E-5
GOTERM_BP_5	Axon guidance	12	6.8E-5
GOTERM_BP_5	Tube morphogenesis	10	7.1E-5
GOTERM_BP_5	Central nervous system development	10	8.5E-5
GOTERM_BP_5	Lipid biosynthesis	20	9.8E-5
GOTERM_BP_5	Monosaccharide metabolism	15	1.1E-4
GOTERM_BP_5	Regulation of biological process	41	1.4E-4
GOTERM_BP_5	Ossification	12	1.5E-4
GOTERM_BP_5	Transcription	99	1.6E-4
GOTERM_BP_5	Carbohydrate catabolism	11	2.2E-4
GOTERM_CC_5	Cell	203	2.6E-4
GOTERM_BP_5	**Neural crest cell development**	6	2.7E-4
GOTERM_BP_5	**Regulation of development**	11	2.9E-4
GOTERM_BP_5	Regulation of cellular process	35	3.1E-4
GOTERM_BP_5	DNA metabolism	34	3.4E-4
GOTERM_BP_5	Neuron morphogenesis during differentiation	16	3.4E-4
GOTERM_BP_5	Cellular macromolecule catabolism	21	3.5E-4
GOTERM_BP_5	Branching morphogenesis of a tube	7	4.1E-4
GOTERM_BP_5	Morphogenesis of a branching structure	7	4.1E-4
GOTERM_BP_5	Neurogenesis	15	4.3E-4
GOTERM_CC_5	Anchored to plasma membrane	5	4.8E-4
GOTERM_CC_5	Anchored to membrane	5	4.8E-4
GOTERM_BP_5	Cellular morphogenesis during differentiation	17	4.9E-4
GOTERM_BP_5	Pattern specification	10	4.9E-4
GOTERM_BP_5	**Skeletal development**	8	5.0E-4
GOTERM_BP_5	Limb morphogenesis	7	6.2E-4
GOTERM_BP_5	Appendage morphogenesis	7	6.2E-4
GOTERM_BP_5	**Appendage development**	7	6.2E-4
GOTERM_BP_5	Regulation of cell differentiation	9	8.5E-4
GOTERM_BP_5	Patterning of blood vessels	6	8.5E-4
GOTERM_BP_5	Vasculogenesis	6	8.5E-4
GOTERM_BP_5	Regulation of myeloid cell differentiation	4	1.1E-3
GOTERM_BP_5	Cellular carbohydrate metabolism	21	1.2E-3
GOTERM_BP_5	Neural crest cell migration	5	1.2E-3
GOTERM_BP_5	Steroid metabolism	14	1.5E-3
KEGG_PATHWAY	Focal adhesion	25	1.5E-3
KEGG_PATHWAY	TGF-β signaling pathway	14	1.7E-3
GOTERM_BP_5	**Exocrine system development**	4	1.8E-3
GOTERM_BP_5	Salivary gland morphogenesis	4	1.8E-3
GOTERM_BP_5	**Salivary gland development**	4	1.8E-3
GOTERM_BP_5	Negative regulation of cell differentiation	6	2.0E-3
KEGG_PATHWAY	ECM-receptor interaction	14	2.1E-3
GOTERM_BP_5	Biomineral formation	7	2.1E-3
GOTERM_BP_5	Ureteric bud branching	5	2.2E-3
GOTERM_CC_5	Intracellular	183	2.3E-3
GOTERM_BP_5	Negative regulation of signal transduction	10	2.3E-3
GOTERM_MF_5	Heparin binding	8	2.4E-3
GOTERM_BP_5	**Ureteric bud development**	6	2.5E-3
GOTERM_BP_5	**Lung development**	7	2.9E-3
GOTERM_BP_5	**Gland development**	4	2.9E-3
GOTERM_BP_5	Mesenchymal cell differentiation	5	2.9E-3
GOTERM_BP_5	**Mesenchymal cell development**	5	2.9E-3
KEGG_PATHWAY	Hedgehog signaling pathway	10	3.4E-3
GOTERM_BP_5	Embryonic appendage morphogenesis	6	3.5E-3
GOTERM_BP_5	**Embryonic development (sensu Metazoa)**	10	3.6E-3
GOTERM_BP_5	Bone remodeling	7	4.9E-3
GOTERM_BP_5	Alcohol biosynthesis	6	4.9E-3
GOTERM_BP_5	Negative regulation of development	6	4.9E-3
GOTERM_MF_5	Nucleic acid binding	8	5.4E-3
GOTERM_CC_5	Transcription factor complex	28	5.4E-3
KEGG_PATHWAY	Glycolysis/gluconeogenesis	10	5.7E-3
GOTERM_BP_5	Neural crest cell differentiation	4	5.8E-3
GOTERM_BP_5	**Cartilage development**	4	5.8E-3
GOTERM_BP_5	Cartilage condensation	4	5.8E-3
GOTERM_BP_5	Axonogenesis	8	5.9E-3
GOTERM_BP_5	Tissue remodeling	7	6.2E-3
GOTERM_BP_5	Regulation of cell migration	7	6.2E-3
GOTERM_BP_5	Neurite morphogenesis	8	6.5E-3
GOTERM_BP_5	Sterol metabolism	8	6.5E-3
GOTERM_BP_5	Cellular morphogenesis	14	7.0E-3
GOTERM_BP_5	Regulation of physiological process	28	7.0E-3
GOTERM_BP_5	Positive regulation of cellular metabolism	18	7.5E-3
GOTERM_BP_5	Regulation of cell motility	7	7.7E-3
GOTERM_BP_5	Hindlimb morphogenesis	4	7.7E-3
GOTERM_BP_5	Carboxylic acid metabolism	27	8.0E-3
GOTERM_BP_5	Carbohydrate biosynthesis	9	8.1E-3
GOTERM_BP_5	Somitogenesis	5	8.1E-3
GOTERM_BP_5	Hemopoiesis	7	8.6E-3
GOTERM_BP_5	**Hemopoietic or lymphoid organ development**	7	9.5E-3
GOTERM_BP_5	Regulation of cellular physiological process	24	9.6E-3
GOTERM_BP_5	**Neuron development**	8	1.0E-2
GOTERM_BP_5	**Respiratory tube development**	6	1.1E-2
GOTERM_BP_5	Embryonic pattern specification	5	1.1E-2
GOTERM_BP_5	**Metanephros development**	6	1.2E-2
GOTERM_BP_5	Cellular lipid metabolism	26	1.3E-2
GOTERM_BP_5	Anterior/posterior pattern formation	5	1.3E-2
GOTERM_BP_5	Protein complex assembly	11	1.5E-2
KEGG_PATHWAY	Pentose phosphate pathway	6	1.5E-2
GOTERM_CC_5	Organelle	179	1.5E-2
GOTERM_CC_5	Intracellular organelle	179	1.5E-2
GOTERM_BP_5	Glial cell differentiation	4	1.6E-2
GOTERM_BP_5	Wnt receptor signaling pathway	10	1.6E-2
GOTERM_CC_5	Membrane-bound organelle	157	1.8E-2
GOTERM_CC_5	Intracellular membrane-bound organelle	157	1.8E-2
GOTERM_MF_5	Catalytic activity	63	1.9E-2
BIOCARTA	Pertussis toxin-insensitive CCR5 signaling in macrophage	5	2.0E-2
GOTERM_BP_5	Genitalia morphogenesis	3	2.1E-2
GOTERM_BP_5	**Placenta development**	3	2.1E-2
GOTERM_CC_5	Nucleoplasm	31	2.1E-2
GOTERM_MF_5	Iron ion binding	16	2.1E-2
GOTERM_BP_5	Positive regulation of biological process	12	2.1E-2
GOTERM_BP_5	Odontogenesis	4	2.3E-2
GOTERM_BP_5	Positive regulation of cell proliferation	10	2.4E-2
KEGG_PATHWAY	Axon guidance	15	2.5E-2
GOTERM_BP_5	Positive regulation of cellular process	10	2.7E-2
GOTERM_BP_5	**Embryonic placenta development**	3	2.8E-2
GOTERM_BP_5	Cell proliferation	11	2.8E-2
GOTERM_BP_5	Negative regulation of cellular process	11	3.0E-2
GOTERM_BP_5	Inner ear morphogenesis	5	3.1E-2
GOTERM_BP_5	Negative regulation of biological process	12	3.2E-2
GOTERM_BP_5	Base-excision repair	4	3.6E-2
GOTERM_BP_5	Negative regulation of neuron differentiation	3	3.7E-2
GOTERM_MF_5	DNA N-glycosylase activity	3	3.7E-2
KEGG_PATHWAY	Fructose and mannose metabolism	8	3.8E-2
GOTERM_BP_5	Amino acid derivative metabolism	7	3.8E-2
GOTERM_CC_5	Nucleosome	7	4.1E-2
GOTERM_BP_5	Positive regulation of cellular physiological process	7	4.1E-2
GOTERM_CC_5	Chromosome	19	4.1E-2
GOTERM_MF_5	Oxidoreductase activity	11	4.2E-2
GOTERM_BP_5	Morphogenesis of an epithelium	5	4.2E-2
GOTERM_MF_5	Protein kinase activity	27	4.6E-2
GOTERM_CC_5	Nuclear lumen	35	4.6E-2
GOTERM_BP_5	Hormone metabolism	4	4.7E-2
GOTERM_MF_5	Pyrophosphatase activity	21	4.8E-2
GOTERM_CC_5	Plasma membrane	17	5.0E-2
GOTERM_BP_5	Regulation of progression through cell cycle	20	5.0E-2
GOTERM_BP_5	Odontogenesis (sensu Vertebrata)	4	5.3E-2
GOTERM_CC_5	Membrane-bound vesicle	8	5.3E-2
GOTERM_CC_5	Vesicle	8	5.3E-2
GOTERM_CC_5	Cytoplasmic vesicle	8	5.3E-2
KEGG_PATHWAY	WNT signaling pathway	15	5.5E-2
GOTERM_CC_5	Protein complex	36	5.5E-2
GOTERM_BP_5	Nucleoside metabolism	4	5.9E-2
GOTERM_BP_5	Segmentation	4	5.9E-2
GOTERM_BP_5	Morphogenesis of embryonic epithelium	4	5.9E-2
GOTERM_BP_5	Sex differentiation	5	6.0E-2
GOTERM_MF_5	Interleukin-11 receptor activity	2	6.1E-2
GOTERM_MF_5	Interleukin-11 binding	2	6.1E-2
GOTERM_CC_5	Chromatin	11	6.2E-2
GOTERM_CC_5	Heterotrimeric G-protein complex	5	6.3E-2
BIOCARTA	Sonic hedgehog (shh) pathway	4	6.4E-2
GOTERM_CC_5	Cytosol	6	6.5E-2
GOTERM_BP_5	Regulation of enzyme activity	4	6.5E-2
GOTERM_CC_5	Intrinsic to Golgi membrane	4	6.6E-2
KEGG_PATHWAY	Biosynthesis of steroids	4	6.8E-2
KEGG_PATHWAY	Huntington's disease	5	6.8E-2
GOTERM_BP_5	**Eye development**	5	7.0E-2
GOTERM_BP_5	Regulation of cell proliferation	9	7.2E-2
GOTERM_BP_5	Biopolymer modification	59	7.2E-2
GOTERM_BP_5	Physiological process	117	7.2E-2
GOTERM_BP_5	Cellular physiological process	100	7.7E-2
GOTERM_BP_5	Astrocyte differentiation	2	7.7E-2
GOTERM_BP_5	Regulation of astrocyte differentiation	2	7.7E-2
GOTERM_BP_5	Mesoderm morphogenesis	3	7.8E-2
GOTERM_BP_5	Mesoderm formation	3	7.8E-2
GOTERM_BP_5	**Mesoderm development**	3	7.8E-2
GOTERM_BP_5	T cell activation	3	7.8E-2
GOTERM_BP_5	**Embryonic heart tube development**	3	7.8E-2
GOTERM_BP_5	Negative regulation of Wnt receptor signaling pathway	3	7.8E-2
GOTERM_BP_5	Negative regulation of BMP signaling pathway	3	7.8E-2
GOTERM_BP_5	M phase of mitotic cell cycle	9	7.8E-2
GOTERM_BP_5	Regulation of signal transduction	8	7.8E-2
GOTERM_BP_5	Neural plate morphogenesis	4	7.9E-2
GOTERM_BP_5	Ear morphogenesis	4	7.9E-2
GOTERM_BP_5	**Ear development**	4	7.9E-2
KEGG_PATHWAY	Synthesis and degradation of ketone bodies	3	8.0E-2
GOTERM_BP_5	**Eye development (sensu Mammalia)**	5	8.6E-2
GOTERM_MF_5	Acyltransferase activity	9	8.7E-2
BIOCARTA	Repression of pain sensation by the transcriptional regulator DREAM	3	8.7E-2
BIOCARTA	IL12 and Stat4 dependent signaling pathway in Th1 development	4	8.8E-2
GOTERM_BP_5	Anatomical structure formation	3	9.0E-2
GOTERM_BP_5	Formation of primary germ layer	3	9.0E-2
GOTERM_MF_5	Bisphosphoglycerate mutase activity	2	9.0E-2
GOTERM_MF_5	Phosphoglycerate mutase activity	2	9.0E-2
GOTERM_BP_5	Phosphate metabolism	35	9.6E-2
BIOCARTA	Rho-selective guanine exchange factor akap13 mediates stress fiber formation	3	9.7E-2
KEGG_PATHWAY	Pyruvate metabolism	6	9.8E-2
KEGG_PATHWAY	Regulation regulation of actin cytoskeleton	18	9.9E-2

*Fold change >2, Student's *t*-test *p *value < 0.01. Count indicates the number of genes in the functional annotation category. The *p *value is from gene enrichment in annotation terms calculated by the Fisher's exact *t*-test.

Genes belonging to GO categories related to the proliferative processes, such as M phase of mitotic cycle and DNA metabolism are specifically downregulated in the BMP2^+ ^cells. Additional data files 6-8 list the genes belonging to the GO 'development' category, TGF-β KEGG pathway and the GO 'M phase' category. The most strikingly downregulated genes from the TGF-β KEGG pathway (Additional data file 7) are *Bmp5*, *Fst *(follistatin), *Id1 *(inhibitor of DNA binding 1) and *Tgfβ2 *(TGF-β). BMPs are members of the TGF-β superfamily of signal molecules, which mediate many diverse biological processes ranging from early embryonic tissue patterning to postnatal tissue homeostasis [30]. WNT, Notch, FGF, Hedgehog and BMP signaling pathways act together during embryogenesis, tissue regeneration and carcinogenesis [31]. Follistatin is a BMP antagonist that regulates the actions of the TGF-β superfamily members [32].

### Selected GO biological process annotations of genes differentially expressed in BMP2^+ ^cells

We analyzed those transcripts that are differentially expressed in BMP2^+ ^cells and involved in selected GO categories of the 'biological process' branch (Additional data file 9). SOURCE [33] was used to obtain GO annotations for the category 'biological process'. The Genesis GO browser (version 1.7.0) [34,35] was used to identify transcripts of interest belonging to the biological process categories adhesion, cell cycle, cell death, cell-cell signaling, cellular metabolism, development, stress response, signal transduction, transcription, and transport. Numbers of these transcripts for each selected category are displayed as separate up- and downregulated groups (Additional data file 9, parts B and D).

More stress-related and less developmental genes are identified when the gene expression levels in undifferentiated ES cells are taken into account ('three condition comparative analysis') than when expression levels are compared between BMP2^+ ^cells and control cells alone (pairwise comparison). In the three condition comparative analysis, all 16 cell death-related transcripts are upregulated in BMP2^+ ^cells. When analyzed further, most of them are apoptosis-related genes. This annotation suggests that cell death during ES cell differentiation mainly involves apoptosis. When ES cells are not taken into account in the two condition comparison, some (18 out of 49) cell death transcripts are downregulated in BMP2^+ ^cells. In reference to signal transduction during BMP2^+ ^cell differentiation, more transcripts are differentially upregulated than downregulated in BMP2^+ ^cells compared to ES cells and control EBs. However, the ratio is reversed when BMP2^+ ^cells are compared only with control EBs. The control EBs differentiat to various cell populations and, thus, more signaling pathways are activated than in BMP2^+ ^cells, which eventually contribute to signaling pathways limited to development.

Hierarchical clustering of genes identified as differentially expressed and involved in development in the pairwise comparison illustrates how transcripts distribute into co-regulated groups and show good reproducibility between experimental replicates (Additional data file 9, part E). Interestingly, the experimental conditions 'BMP2^+ ^cells' and 'undifferentiated cells' are more closely related to each other than to the condition 'control EBs', indicating an earlier developmental stage of BMP2^+ ^cells compared to control EBs of the same age (Additional data file 9, part E).

### Expression of genes in the BMP2^+ ^cells associated with plasticity, and mesodermal and NCSC phenotypes

#### BMP2^+ ^cells are still in a state of plasticity

**Figure 2:**
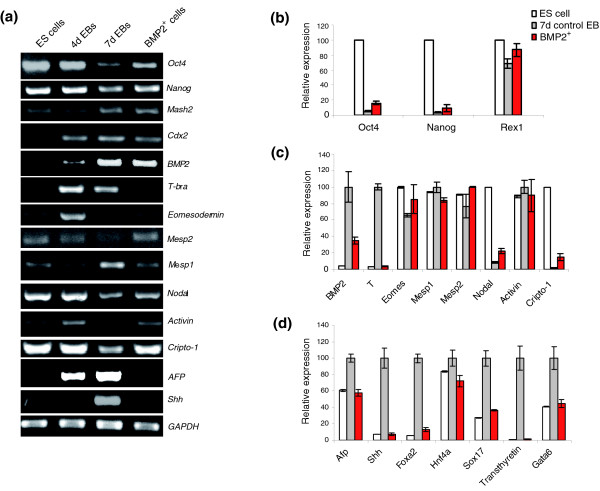
Expression of pluripotent, trophectodermal, mesodermal, and endodermal gene markers in BMP2^+ ^cells. **(a) **RT-PCR analysis for the representative genes. **(b-d) **Relative expression level of the genes presented in (a) and additional representative genes as obtained by Affymetrix analysis. The expression levels of each gene were normalized with its maximum level set as 100%. Each result was an average of three independent experiments (Additional data file 13).

BMP2^+ ^cells significantly upregulate Oct4 and Nanog transcript expression compared to the control EBs, in which several somatic cell types develop (Figure 2a,b), but at a level lower than ES cells. This implies that there are some populations of BMP2^+ ^cells with multi-lineage progenitor phenotypes, which are still in a certain state of plasticity and can give rise to different cell fates depending upon the stimuli. This is further confirmed by the upregulated expression of leukemia inhibitory factor (LIF) in the BMP2^+ ^population compared to the control EBs. Interestingly, the transcripts of Activin, Nodal and Cripto are also upregulated in the BMP2^+ ^population compared to the control EBs. Recently, it has been demonstrated that the TGF-β/Activin/Nodal signaling pathway is necessary for the maintenance of pluripotency in ES cells [36]. Therefore, the increased levels of the pluripotency associated gene markers Oct4 and Nanog might be explained, in part, by the increased expression of LIF, Activin and Nodal observed in the BMP2^+ ^cells (Figure 2). It is worth mentioning the hypothesis by Niwa *et al*. [37] that to maintain the undifferentiated stem-cell phenotype, Oct-3/4 expression must remain within plus or minus 50% of normal diploid expression. If Oct-3/4 expression is increased beyond the upper threshold level, differentiation into primitive endoderm or mesoderm is triggered. If Oct-3/4 expression is decreased, stem cells are redirected into the trophectoderm lineage. This partly explains the increased levels of Oct-3/4 by the BMP2^+ ^mesodermal lineages [37].

#### The BMP2^+ ^cells exhibit mesodermal characteristics

The expression levels of nodal, activin, eomesodermin, cripto and mesoderm posterior 2 (Mesp2) was increased in BMP2^+ ^cells whereas the expression of T-bra and Mesp1 was lower in the BMP2^+ ^cells compared to the control EBs (Figure 2). It has been shown that Activin and Nodal, members of the TGF-β superfamily, play pivotal roles in inducing and patterning mesoderm and endoderm, as well as in regulating neurogenesis and left-right axis asymmetry (for a review, see [38]). Nodal genes have been identified in numerous vertebrate species and are expressed in specific cell types and tissues during embryonic development [38]. Moreover, Nodal null mouse mutants lack mesoderm. Overexpression of Nodal in mouse ES cells leads to upregulation of mesodermal and endodermal cell markers. These findings support the key role of Nodal for mesoderm formation [39]. Also, it was repeatedly shown that Activin is involved in the mesodermal pattering during *Xenopus *embryo development [40]. Cripto is the founder member of the Cripto/FRL-1/Cryptic (CFC) family. Cripto is expressed in tumor tissues, and studies in the mouse have established an essential role for cripto in the formation of precardiac mesoderm and differentiation into functional cardiomyocytes [41].

The T-box gene encoding eomesodermin (*Eomes*) is required for mesoderm formation and the morphogenetic movements of gastrulation. Lack of *Eomes *abrogates the formation of embryonic and extra-embryonic mesoderm [42]. It has been shown that *Eomes *is specifically required for the directed movement of cells from the epiblast into the streak in response to mesoderm induction [42]. Interestingly, we found a dramatically low level of T-Brachyury expression in BMP2^+ ^cells compared to the control EBs. This result suggests that the BMP2^+ ^cells represent a subset of a mesodermal population whose formation is nearly complete at the time of BMP2 expression, which in turn downregulates T-Brachyury expression. However, this hypothesis does not rule out the possibility that the necessary signals from the non-BMP2 population to induce stronger T-Brachyury expression are eliminated due to puromycin selection. T-Brachyury is an essential gene for the mesoderm formation, as demonstrated in the mouse [43]. Mesp1 and Mesp2 are basic helix-loop-helix transcription factors that are co-expressed in the anterior presomitic mesoderm just prior to somite formation in the mouse embryo [44]. Furthermore, it has been shown that Mesp1 has a significant role in the epithelialization of somitic mesoderm and, therefore, it is assumed that Mesp2 is responsible for the rostro-caudal patterning process itself in the anterior presomitic mesoderm [44]. Recently, it has been shown that Mesp1 is expressed in almost all of the precursors of the cardiovascular system in the mouse, including the endothelium, endocardium, myocardium and epicardium [45]. Thus, the *in vitro *derived BMP2^+ ^cells exhibit more mesodermal characteristics. This conclusion is further supported by the derivation of most of the mesodermal tissues and complete absence of endodermal phenotypes from these BMP2 cells in the later stages, as described below. Interestingly, Noggin, an antagonist of BMP2, is also expressed in the BMP2^+ ^cells at a level higher than in ES cells but lower than in the control EBs. This contradiction might be explained on the basis that mesodermal cells exress Noggin and its expression is regulated by BMP2 [46,47]. Also, co-expression of Noggin and glial fibrillary acidic protein (GFAP) in astrocytes has been reported [48].

#### BMP2^+ ^cells lack endodermal phenotypes

Interestingly, transcripts of α-feto protein (AFP) and Sonic hedgehog (Shh) were not detectable in BMP2^+ ^cells (Figure 2). AFP is a marker for the endoderm-derived hepatocytes. The expression pattern of Shh studied in several species indicates that Shh is essential for endoderm-derived organ development, such as foregut, gut, and gastrointestinal duodenal and pancreas development [49]. Also, other gene markers for endoderm, such as Foxa2, Hnf4a, Sox17, Transthyretin and Gata6 [50-54], are downregulated in the BMP2^+ ^cells. These results show a dramatically reduced level or, more likely, the complete absence of the endodermal cell lineage.

### The BMP2^+ ^cell lineage contains neural crest stem cells and their derivatives

**Figure 3:**
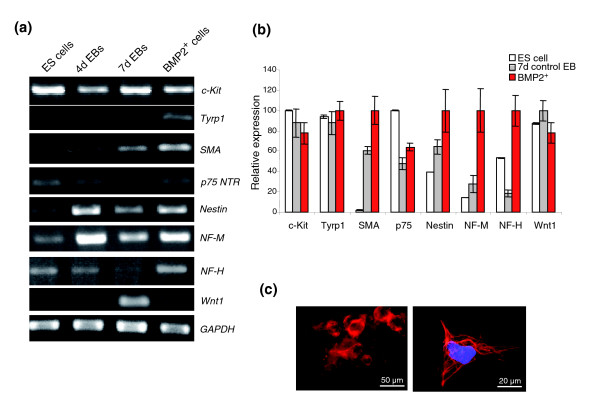
Analysis of neural crest stem cell associated transcripts in BMP2^+ ^cells. **(a) **RT-PCR analysis for the representative genes associated with NCSCs. **(b) **Relative expression level of the genes presented in (a) as obtained by Affymetrix analysis. The expression levels of each gene were normalized with its maximum level set as 100%. Each result is an average of three independent experiments (Additional data file 13). **(c) **Detection of p75 and GFAP in BMP2^+ ^cells labelled by immunohistochemistry. Immunuostainings with anti-p75 (left panel) and anti-GFAP (right panel) to show the presence of NCSCs and the astrocytes, respectively, in BMP2^+ ^cells one day after plating (7 + 1 days in total).

**Figure 4:**
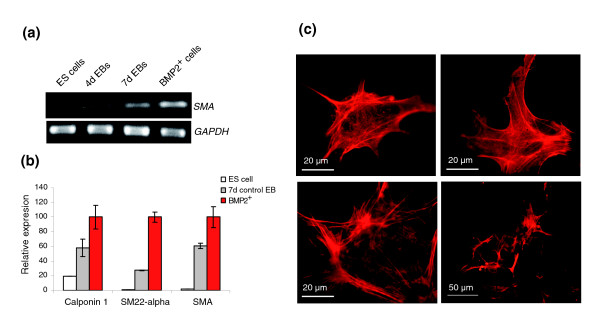
Detection of smooth muscle cells after differentiation of the BMP2^+ ^cells. **(a) **Expression of SMA in the BMP2^+ ^cells detected by qRT-PCR. **(b) **Microarray relative expression levels of various smooth muscle specific genes in BMP2^+ ^cells. The expression levels of each gene were normalized with its maximum level set as 100%. Each result is an average of three independent experiments (Additional data file 13). **(c) **Detection of smooth muscle cells 1 day after plating the BMP2^+ ^cells (7 + 1 days in total; top left), 8 days after plating with puromycin (7 + 8 days in total; top right), 18 days after plating with puromycin (7 + 18 days in total; bottom left) and 18 days after plating without puromycin (7 + 18 days in total; bottom right).

The BMP2^+ ^cells showed enriched expression of ectodermal markers neurofilament (NF)-H and NF-M, but it is evident that the BMP2^+ ^cells shared more mesodermal characteristics as described in the previous sections. Further investigation of this contradictory phenomenon led to the conclusion that these ectodermal markers may be more likely expressed by NCSCs. In agreement with our results, it was repeatedly reported that NCSCs share more ectodermal and less mesodermal characteristics [55-58]. Expression of the NCSC-specific p75^NTR ^and Nestin transcripts at higher levels compared to the control EBs (Figure 3a,b) confirmed the increased presence of NCSCs in BMP2^+ ^cells. In addition, astrocyte-specific GFAP and melanocyte-specific tyrosine phosphatase 1 (Tyrp1) in BMP2^+ ^cells (Figure 3a) confirmed the presence of NCSC-derived lineages in the BMP2^+ ^lineage as well. Expression of p75^NTR ^in BMP2^+ ^cells was further confirmed by immunostaining with an antibody against p75^NTR ^in BMP2^+ ^cells (Figure 3c, left panel). Furthermore, the presence of glial cells in BMP2^+ ^cells has been confirmed by immunostaining with an antibody against GFAP (Figure 3c, right panel). Notably, it has been reported that the differentiation of NCSCs into their lineage fates is mainly dependant on the presence of BMP2 at the required threshold level and also the availability of other factors, such as TGFβ1, Wnt1, Ihh and BMP4, in combination [56]. It was well demonstrated that exogenous addition of recombinant BMP2 to cultured NCSCs isolated from chicken explants of cranial and trunk dorsal neural folds from stage 8/9 embryos resulted in the differentiation of NCSC into glia, melanocytes and smooth muscle cells [56]. Expression of smooth muscle α actin (SMA) was also detected in the differentiated BMP2^+ ^cells (Figure 4).

It is surprising to note that the local concentration of BMP2 secreted by BMP2^+ ^cells themselves is able to induce the differentiation of NCSCs into their lineages without the exogenous addition of BMP2.

During vertebrate embryonic development, when the notochord induces the transformation of surface ectoderm to neuroectoderm, a multipotential middle cell layer develops with characteristics of both cell types. These cells are the neural crest cells. They migrate dorsolaterally to form the neural crest, a flattened irregular mass between the surface ectoderm and neuroectoderm. This layer of cells separates into right and left portions and then migrates to various locations within the embryo to give rise to most structures of the peripheral nervous system, such as Schwann and glia cells of the autonomic and enteric nervous systems, endocrine cells, such as the adrenal medulla, and C-cells of the thyroid as well as non-neural tissues, such as pigment cells of the skin and internal organs, smooth muscle of the cardiac outflow tract and great vessels, pericytes, craniofacial bones, cartilage and connective tissues [59,60].

Wnt1 and c-kit are well known mediators of melanocyte differentiation of NSCS [61,62] and are found to be expressed in the BMP2^+ ^population. The tyrosine kinase c-kit has been found in the cell membranes of haematopoietic stem cells, primordial germ cells and presumptive subepidermal melanocytes [63]. Intriguingly, wnt-1 and BMP2 act synergistically to suppress differentiation and to maintain NCSC marker expression and multipotency by combinatorial Wnt1/BMP2 signaling [64].

The presence of Wnt1 transcripts in the BMP2^+ ^cell population may be interpreted in two ways: first, Wnt1 may be involved in the maintenance of NCSCs in their pluripotency state in combination with BMP2; and second, Wnt1 may be involved in driving the differentiation of NCSCs into melanocytes. Both possibilities cannot be ruled out in the BMP2^+ ^cell population since it includes proliferating NCSCs on the one hand (increased cell number when subjected to immunostaining) and melanocytes in the same culture on the other hand. The local BMP2 and/or Wnt1 gradient may drive the NCSCs to produce smooth muscle cells, pericytes, or melanocytes or to remain in their pluripotent state, respectively.

This is the first study that enables us to selectively obtain ES cell-derived NCSCs and their derivatives all at the same time via a *BMP2 *promoter-based lineage selection approach. The co-expression of Wnt1 and BMP2 indicates the existence of an environment to both keep the NCSCs in stemness and to enable ongoing differentiation of NCSCs to form melanocytes. Thus, the study of these BMP2-expressing cells during early differentiation of ES cells will pave the way for a better understanding of NCSCs and their differentiation into lineages. The BMP2^+ ^cells derived from the ES cells may serve as an ideal model for neural crest stem biology in the future since the NCSCs and their derivatives can be selectively enriched by the *BMP2 *promoter-driven lineage selection approach. In addition, it provides a valuable system where the enriched NCSCs prime themselves to differentiate into their cell specific lineages, since the enriched NCSCs secrete BMP2 and cause a BMP2 gradient, which negates the need for supplying exogenous BMP2. Noggin, an inhibitor of BMP2, can be used to keep the NCSCs in a plastic state. Once the procedures for maintaing BMP2-derived NCSCs in a state of plasticity have been fine tuned, they will be potential candidates for cell replacement therapy, since they can differentiate into any tissue depending upon the local environment of the tissue in which they are injected.

### BMP2^+ ^cells contain predominantly smooth muscle cells

As indicated in Figure 4a, expression of SMA in the BMP2^+ ^cells is enriched compared to the control EBs. In addition, the microarray data confirm the upregulation of SMA and other smooth muscle specific genes, such as those encoding calponin and SM22-α, in the BMP2^+ ^cell population compared to control EBs (Figure 4b). The immunostaining of smooth muscle cells has been performed with an antibody against SMA, 1 day after plating the BMP2^+ ^cells (7 + 1 days in total), 8 days after plating with puromycin (7 + 8 days in total), 18 days after plating with puromycin (7 + 18 days in total) and 18 days after plating without puromycin (7 + 18 days in total) (Figure 4c). Detection of smooth muscle cells even 18 days after puromycin treatment indicates that smooth muscle cells express BMP2 and, therefore, survive the puromycin treatment [65]. It is noteworthy that the number of SMA positive cells was less in the culture in which puromycin treatment continued compared to the culture in which puromycin treatment was discontinued. The inhibitory effect in these cultures is more likely due to BMP2 since recent reports demonstrated that BMP2 inhibits proliferation of smooth muscle cells [66,67]. Simultaneously, the formation of smooth muscle cells from their precursors is crucially dependant on the phenotypic inductive role of BMP2 [68].

### BMP2^+ ^cells give rise to cardiomyocytes under EB conditions

**Figure 5:**
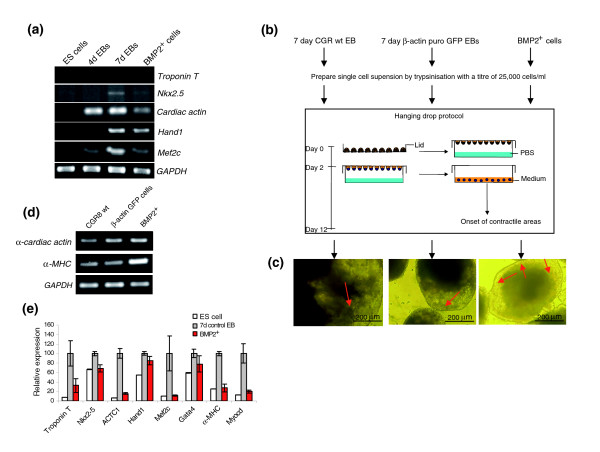
Differentiation of the BMP2^+ ^cells to cardiac cells. **(a) **RT-PCR analysis of the cardiac markers in BMP2^+ ^cells and other controls. **(b) **Schematic outline of the protocol used to derive cardiomyocytes from BMP2^+ ^cells. **(c) **The morphology of the contracting EBs. The red arrows indicate the contracile area(s) in that EB. **(d) **RT-PCR analysis of the representative cardiac markers in 12 day secondary EBs derived by the hanging drop protocol from single cell suspension obtained from seven-day-old primary EBs generated by the protocol outlined in Figure 1 from CGR8 wild-type EBs without puromycin treatment, β-actin puro EGFP EBs and BMP2 EBs with puromycin treatment (videos of the beating clusters in these populations are provided as Additional data files 10-12). **(e) **Graph showing the relative expression levels of the genes presented in (a) as obtained by Affymetrix analysis. The expression levels of each gene were normalized with its maximum level set as 100%. Each result is an average of three independent experiments (Additional data file 13).

The expression of the cardiac marker genes *NKx2.5*, *MLC-2a*, *α-cardiac actin *and *Mef2c *is reduced in the BMP2^+ ^cells compared to the control EBs (Figure 5a,e). This suggests that cardiomyogenesis is repressed in the BMP2^+ ^cells. Accordingly, Mesp1 was repressed (Figure 2a).

EBs prepared from a mixture of BMP2^+ ^cells with wild-type ES cells in ratios of 1:1, 1:2, 1:4, 10:1, 50:1, 1:10 and 1:50, respectively (using the hanging drop protocol and applying the differentiation protocol as outlined in Figure 5b and also previously described [69]), did not augment/delay the onset of contractile activity in comparison to control wild-type EBs as observed on day 12. Also, there were no significant differences in terms of the magnitude of the intensity of the contractility (data not shown). This corresponds to the observation that BMP2 added during the differentiation of ES cells did not enhance cardiomyogenesis [70]. Compared to the CGR8 wild-type and β-actin control cells, culturing of the BMP2^+ ^cells for even a further 28 days (35 days in total) in the presence or absence of puromycin did not result in beating clusters of cardiac cells (data not shown). These results suggest that the plated BMP2^+ ^cells did not contain mature beating cardiomyocytes at this stage. In order to investigate the cardiomyogenic potential of the BMP2^+ ^cells to differentiate into cardiac beating cells, EBs were made from the BMP2^+ ^cells using the hanging drop protocol and the differentiation process was observed in comparison to the EBs formed by cells from control EBs. The secondary EBs made from the BMP2^+ ^cells were contracting on day 11, similarly to the secondary EBs formed by cells from seven-day-old control CGR8 wild-type EBs that were not treated with puromycin, as well as EBs made from the β-actin CGR8 clone transfected with *β-actin *promoter-driven puromycin resistance and EGFP expression cassettes that were treated with puromycin in the same way as the secondary BMP2^+ ^EBs. Notably, the intensity of contraction in the BMP2^+ ^EBs was significantly stronger compared to that in both controls (Additional data file 10). The whole EB was contracting in a jellyfish-like fashion compared to the controls (Figure 5c; Additional data files 11 and 12). The contracting areas persisted for more than a week, which was longer than in the control EBs. In order to investigate whether the increased beating activity of the cardiomyocytes generated from the BMP2^+ ^cells correlates with increased expression of cardiomyocyte specific transcripts, we determined the expression of α-cardiac actin and α-MHC in secondary EBs at day 11. Increased expression levels of both cardiac specific genes was observed in the EBs generated from BMP2^+ ^cells compared to both the control secondary EBs (Figure 5d). Thus, BMP2^+ ^cells have the capacity to develop into cardiomyocytes. Interestingly, the cardiomyogenic potential apparently seems to be regulated by the BMP2^+ ^lineage cells only. These findings suggest that the BMP2^+ ^cells are primed to become cardiomyocytes independently of the other, BMP2 negative lineage cells. However, neither the secondary EBs maintained on puromycin nor the contractile secondary EBs when treated with puromycin contained cardiomyocytes. The question of whether the cardiomyocyte precursors contained in the BMP2^+ ^cell population are the transient derivatives of the NCSCs or another cell lineage needs to be investigated, since NCSCs are also capable of differentiating into cardiomyocytes [59,60]. However, although the BMP2^+ ^cells are capable of differentiating into beating cardiomyocytes, further extensive investigations are required to demonstrate the cardiogenic potential of these cells in detail, as previously described for ES cell-derived cardiomyocytes [71].

### Vascular and haematopoietic cell gene markers from the BMP2^+ ^cell population

**Figure 6:**
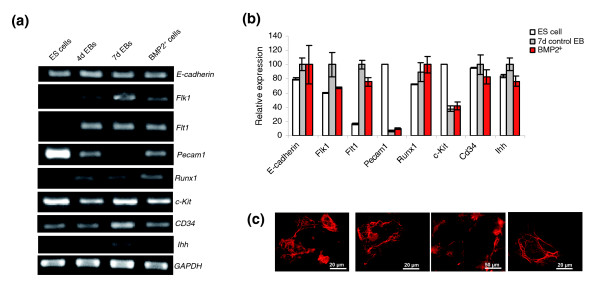
Analysis of the vascular and haematopoietic cell gene markers in the BMP2^+ ^cell population. **(a) **RT-PCR analysis of the BMP2^+ ^cells. **(b) **Relative expression levels of the genes presented in (a) as obtained by Affymetrix analysis. The expression levels of each gene were normalized with its maximum level set as 100%. Each result is an average of three independent experiments (Additional data file 13). **(c) **Immunuostainings with anti-pan cytokeratin over the period of time to show the presence of epithelial like cells, from left to right: one day after plating the BMP2^+ ^cells (7 + 1 days in total); 8 days after plating with puromycin (7 + 8 days in total); 18 days after plating with puromycin (7 + 18 days in total); and 11 days after plating without puromycin (7 + 11 days in total).

Expression of E-cadherin, Flk1, Flt1, Pecam1 and Runx1 shows the presence of vascular endothelial progenitors in the BMP2^+ ^population (Figure 6a). As demonstrated in Figure 1j, BMP2^+ ^cells are able to differentiate into cells with an epithelial/endothelial morphology after being cultured for eight days in the presence of puromycin. Detection of epithelial-like cells has been confirmed by immunostaining. As indicated in Figure 6c, epithelial-like cells were detected even after 11 days of culturing in the presence of puromycin.

Expression of c-kit and CD34 and Runx1 indicates the presence of haematopoietic stem cells in the BMP2^+ ^population (Figure 6a,b). *Ihh*, which is implicated in haematopoiesis and vasculogenesis [72] is also expressed in the BMP2^+ ^cells but its expression level is lower than that of the control cells. Flt-1, also known as vascular endothelial growth factor receptor 1 (VEGFR-1), is a high-affinity tyrosine kinase receptor for vascular endothelial growth factor and is normally expressed only in vascular endothelial cells. However, the Flt-1 transcript was recently found to be expressed in human peripheral blood monocytes [73]. Monocytes are known to differentiate into a variety of cell types, such as osteoclasts in bone, dendritic cells in the immune system and mature macrophages in a number of tissues, for example, Kupffer cells in liver [73]. Expression of Flt-1 and c-fms in the BMP2^+ ^population indicates the possible existence of a monocyte-macrophage lineage in these cells.

### BMP2^+ ^cells give rise to osteoblasts and express gene markers for satellite cells and fibroblasts

**Figure 7:**
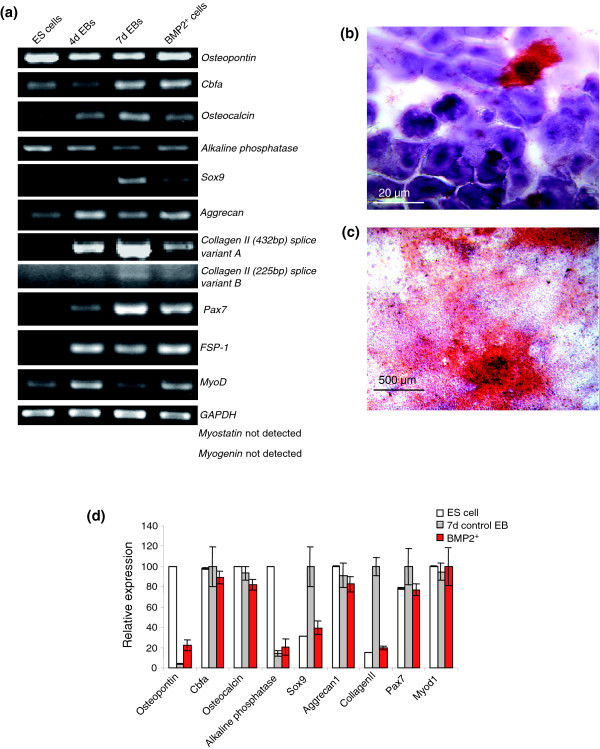
Analysis of osteoblast, chondrocyte and myocyte specific markers in BMP2^+ ^cells. **(a) **RT-PCR analysis in BMP2^+ ^cells. **(b,c) **Alizarin stanings on the 18th day after plating the BMP2^+ ^cells (7 + 18 days in total) and 28 days after plating (7 + 28, 35 days in total). **(d) **Relative expression levels of the genes presented in (a) as obtained by Affymetrix analysis. The expression levels of each gene were normalized with its maximum level set as 100%. Each result is an average of three independent experiments (Additional data file 13).

Expression of osteopontin, Cbfa, osteocalcin and alkaline phosphatase in BMP2^+ ^cells showed the possible occurrence of osteoblasts (Figure 7a). BMP2^+ ^cells plated and maintained in the differentiation medium with puromycin showed Alizarin red positive clusters after 18 days (Figure 7b), which indicates the first appearance of osteoblasts. After 35 days of culturing in the presence of puromycin, areas brightly stained with Alizarin red were observed, confirming the occurrence of osteoblasts in culture (Figure 7c). These results show that the puromycin resistant BMP2^+ ^cells *per se *are capable of differentiating into osteoblasts. It may also be possible that the osteoblasts might develop from the BMP2^+ ^derived NCSCs in the presence of an increased BMP2 concentration for longer periods of time [74].

Expression of the transcription factor Sox9, the proteoglycan aggrecan, and collagen II (with two alternative splicing forms) was detected in the BMP2^+ ^population, indicating the existence of chondrocytes [75] (Figure 7a). Sox9 is a key transcriptional factor for chondrocytic differentiation of mesenchymal cells via chondrocyte-specific enhancer of the pro alpha1(II) collagen [76]. Expression of Pax-7 and myoD shows the presence of satellite cells [77,78]. Satellite cells represent a distinct lineage of myogenic progenitors responsible for the maintenance of skeletal muscle [79]. MyoD is expressed only when satellite cells are activated to proliferate and differentiate into primary myoblasts, which will in turn differentiate into cells of the myofibres of skeletal muscles [78]. An elevated level of MyoD indicates either the proliferation or the differentiation of satellite cells into primary myoblasts in the BMP2^+ ^population. But the expression of myogenin and myostatin was not detected by RT-PCR, even after 40 cycles in the BMP2^+ ^cell population, suggesting the absence of skeletal muscle cells. This hypothesis is supported by Affymetrix data that show that myogenin is very weakly expressed; myostatin (*Gdf8*) is not present on the Affymetrix chip.

Interestingly, it has been reported that the satellite cells can spontaneously differentiate into adipocytes in an alternative mesenchymal pathway [80]. Expression of FSP-1 shows the occurrence of fibroblasts in the BMP2^+ ^cell population. FSP-1 has been implicated in the epithelial-mesenchymal transition and is a representative marker for mesenchymal cells [81].

### The BMP2^+ ^cell lineage contains monocytes but not mature adipocytes

**Figure 8:**
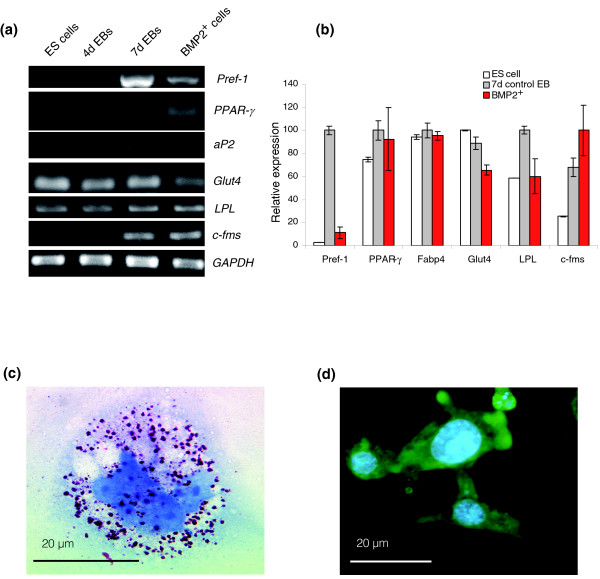
Analysis of adipocyte and macrophage associated markers in BMP2^+ ^cells. **(a) **RT-PCR analysis of adipocyte specific markers. **(b) **Relative expression levels of the genes presented in (a) as obtained by Affymetrix analysis. The expression levels of each gene were normalized with its maximum level set as 100%. Each result is an average of three independent experiments (Additional data file 13). **(c) **Sudan red staining on 11-day-old puromycin untreated culture after plating the BMP2^+ ^cells (7 + 11, 18 days in total). **(d) **F4/80 immunuostaining on 18-day-old puromycin untreated culture after plating BMP2^+ ^cells (7 + 18, 25 days in total).

The RT-PCR analysis of the BMP2^+ ^population showed the expression of the pre-adipocyte gene marker pref-1 [82], the pan-adipocyte markers PPARγ [83], aP2, Glut4 [84] and LPL as well as the pan-macrophage marker c-fms [82,83,85] (Figure 8a,b). Although the markers PPARγ, aP2 and Glut4 are unique to adipocytes, these genes are also expressed in macrophages. Similarly, the trophoblast lineage also expresses c-fms [82]. Notably, the cells derived from the BMP2^+ ^cells cultured in the continual presence of puromycin were not positive for either Sudan Red or anti F4/80 stainings. However, when puromycin treatment was discontinued, there were cells stained with Sudan Red and F4/80, a marker for macrophages, as shown in Figure 8c,d. These findings suggest that macrophages are derived from differentiated BMP2^+ ^cells in which BMP2 expression is downregulated at a later time. There were no cells stained with Oil Red O in both cases (with discontinued puromycin treatment), even when cultured for a prolonged period of days. Thus, the BMP2^+ ^population might contain the pre-adipocytes expressing only PPARγ, aP2, Glut4 and Pref-1 but not adipsin. Apparently, other factors secreted by BMP2 negative cells are required for maturation of pre-adipocytes to adipocytes.

## Conclusion

The *in vitro *ES-derived BMP2^+ ^population contains trophectoderm, NCSCs and their derivatives - smooth muscle cells, pericytes, melanocytes, cardiomyocytes as well as vascular and haematopoietic progenitors. Enrichment of the neural crest cells and their lineages by *BMP2 *promoter-driven selection markers paves the way for ES cell-derived neural crest stem cell biology. These cells are attractive candidates for future cell replacement therapies due their ability to diferentiate into any tissue depending upon the local environment within the injected tissue. Furthermore, we showed that the increased developmental potential of the BMP2^+ ^expressing cells is accompanied by increased transcriptional and apoptotic activity. Finally, the identification of the genes that are specifically expressed in the BMP2^+ ^population will contribute to a gene expression atlas for mesodermal developmental genes that will be useful for further studies to elucidate their role during developmental processes.

## Materials and methods

### ES cell culture and differentiation of EBs

Murine CGR8 ES cells (ECACC 95011018) were cultured in the absence of feeder cells in Glasgow minimum essential medium (GMEM) supplemented with 10% fetal bovine serum (FBS), 2 mM L-glutamine, 100 units/ml leukemia inhibitory factor (LIF) and 50 μM β-mercaptoethanol (ME) in 0.2% gelatine coated flasks as previously described [69]. To induce differentiation, an ES cell suspension of 1.6 × 10^4 ^cells/ml was made in Iscove's modified Dulbecco's Medium (IMDM) supplemented with 20% (FBS), 1% non-essential amino acids (vol/vol), 2 mM L-glutamine and 100 μM β-ME. The cells were cultured in bacteriological dishes for two days, then the two-day-old EBs were transferred into a 0.2% gelatine coated 10 cm tissue culture dish. On day 4, the EBs were treated with 3 μg/ml puromycin for the next 3 days. Medium containing puromycin was refreshed every day. On day 7, the puromycin resistant BMP2^+ ^cells were used for the experiments.

The hanging drop differentiation protocol as described in Figure 1 was carried out as follows. Briefly, 500 undifferentiated BMP2^+ ^ES cells were spotted in 20 μl differentiation medium in the upper lid of a bacteriological dish and cultured for 2 days. On day 2, the EBs were transferred into suspension until assays were performed, with medium changes in between as outlined in Figure 5b.

### Vector construct and cell line generation

pIRES2 EGFP was purchased from Clontech (Heidelberg, Germany). Human cytomegalovirus (CMV) immediate early promoter and enhancer were removed by a double digestion with *Nhe*I and *Ase*I and then subsequently blunt end ligated to get the pIRES2 EGFP φ CMV construct. Puromycin cDNA flanked on both ends by *Bam*H1 restriction sites, which was PCR amplified from pIRESPuro3 by *Pfu *DNA polymerase (Promega, Mannheim, Germany), was inserted at the *Sma*I site of the pIRES2 EGFP φ CMV construct to get pPuro IRES2 EGFP. A 2.9 kb *BMP2 *promoter fragment with both proximal and distal transcription start sites, excised with *Hin*dIII and *Sac*I digestion from pBMP2-GL3 [86], were blunt end ligated to *Eco*RI digested and klenowed pPuro IRES2 EGFP to generate pBMP2^p ^Puro IRES2 EGFP. This BMP2 reporter construct drives the expression of both puromycin resistance and EGFP under the control of the *BMP2 *promoter by the use of an IRES sequence. This construct was electroporated in CGR8 with 500 μF and 240 V in a Bio-Rad Gene Pulser™ (Bio-Rad, Hercules, CA, USA). The transfected clones were selected using 400 μg/ml neomycin and, after selection, were maintained with 200 μg/ml neomycin. During EB generation, the neomycin selection was discontinued.

### Immunohistochemistry

One day prior to the sample processing, 50,000 cells per each well of Lab-Tek Permanox slide Chambers (Nalge Nunc International, Naperville, IL, USA) were seeded and cultured in the absence of puromycin. After 24 hours, the samples were fixed with either 4% paraformaldehyde in phosphate-buffered saline for 10 minutes or -20°C precooled methanol-acetone (1:1) solution, permeabilized with 0.1% Triton X-100, and labeled with the following antibodies: mouse anti-SMA (1:200) (Sigma, Taufkirchen, Germany); mouse anti-pan cytokeratin (1:50) (DakoCytomation, Hamburg, Germany); mouse anti-Ksp-cadherin (Zymed, South San Francisco, CA, USA); mouse anti-myoD1 (Santa Cruz, Heidelberg, Germany); anti-actinin antibodies (Sigma, Taufkirchen, Germany); mouse anti-GFAP (1:100) (Sigma, Taufkirchen, Germany); rabbit anti-p75 NTR (1:100), (Chemicon, Hampshire, UK); rabbit anti-S100 (1:400), (DakoCytomation, Hamburg, Germany); and mouse anti-actinin (1:100). This was followed by labeling with secondary antibodies, either Cy3-labeled anti-rabbit IgG (Sigma), rhodamine labeled anti-mouse IgG (Sigma), or Cy3-labeled donkey anti-rat IgG antibodies. The specificity of the antibodies has been tested using the appropriate tissues (Additional data file 13). For a control of SMA immunostaining, vessels of mouse liver were stained. Heart and skeletal muscles of embryonic mouse tissues served as controls for the specifity of the anti-actinin and the myoD1 antibodies, and murine brain tissue for the specifity of antibodies against neuronal markers such as p75-NTR and GFAP. Mouse kidney and small intestine were used as control tissues for Ksp-cadherin or E-cadherin and pan-cyokeratin staining, respectively.

### Sudan Red and Alizarin Red S stainings

Sudan Red staining was performed on paraformaldehyde fixed embryonic cell clusters or on cytospins of human lymphocytes. The paraformaldehyde-fixed samples were treated with Alizarin Red S for five minutes and then excess Alizarin Red was washed off by dipping in acetone:xylene (1:1), followed by a wash in xylene. They were then stained for haematoxylin for ten minutes and the excess dye was washed off in running water.

### Semiquantitative RT-PCR analysis

Total RNA was extracted using RNeasy Mini Kit (Qiagen, Hilden, Germany) with on-column DNase I (Qiagen) digestion according to the manufacturer's instructions. Total RNA (5 μg) was reverse transcribed using SuperScript II Reverse transcriptase (Invitrogen GmbH, Karlsruhe, Germany) with random primers according to the manufacturer's recommended protocol. PCR amplification was done with REDTaq ReadyMix (Sigma) with 0.4 μM of each primer. GAPDH was used as an internal control. The following conditions were used. An initial denaturation at 95°C for 2 minutes, followed by 22-35 cycles of 30 s denaturation at 95°C, 30 s annealing at 60°C and 60 s of elongation at 72°C. A final extension at 72°C for 5 minutes was included. Electrophoretic separation of PCR products was carried out on 2% agarose gels with 0.001% ethidium bromide. The primer pairs included in this study are listed in Additional data file 14.

### Flow cytometry

A single cell suspension was prepared by trypsinization. Cell clumps were removed by passing through the cell strainer cap of a round bottom tube from BD Falcon^® ^(Heidelberg, Germany). Propidium iodide staining (Sigma) was included to exclude dead cells. Acquisition of 10,000 live (PI negative) cells was done with a FACScan (BD Biosciences, Heidelberg, Germany) and the data analysis was done with CellQuest software (Becton Dickinson, Heidelberg, Germany). The wild-type EBs were used as the control on the same day as the sample EBs.

### Affymetrix analysis

Total RNA was extracted from undifferentiated ES cells and EBs using the RNeasy total RNA isolation kit (Qiagen GmbH, Hilden, Germany). The preparation quality was assessed by agarose formaldehyde gel electrophoresis. Three independent total RNA preparations, each 15 μg from the BMP2^+ ^cells, the mixed cell population and the undifferentiated ES cells, were labelled with the One-Cycle Target Labeling and Control Reagent package (Affymetrix, High Wycombe, UK) as described in the manufacturer's instructions. Briefly, double-stranded cDNA was synthesized using the one-cycle cDNA synthesis module. Biotinylated cRNA was synthesized with the IVT labeling kit and cleaned up using the sample cleanup module.

After fragmenting of the cRNA for target preparation using the standard Affymetrix protocol, 15 μg fragmented cRNA was hybridized for 16 h at 45°C to Mouse Genome 430 2.0 arrays, which carry probe sets representing 45,101 probe sets. Following hybridization, arrays were washed and stained with streptavidin-phycoerythrin in the Affymetrix Fluidics Station 450 and further scanned using the Affymetrix GeneChip Scanner 3000 7G. The image data were analyzed with GCOS 1.4 using Affymetrix default analysis settings. After RMA normalization [87], three pair-wise comparisons were performed using the Student's *t*-test (unpaired, assuming unequal variances). A Student's *t*-test *p *value < 10^-2 ^and a fold change >2 were used to identify and restrict the number of differentially expressed genes. Hierarchical clustering was performed for an intersection of genes differentially expressed between undifferentiated BMP2 ES cells and BMP2^+ ^EBs as well as differentially expressed between seven-day-old control EBs and BMP2^+ ^EBs (three condition comparative analysis) to determine and differentiate treatment and developmental aspects. The cluster analysis (see Additional data file 9, part E) was done using cluster version 2.11 [88], applying mean-centering and normalization of genes and arrays before average linkage clustering with uncentered correlation.

### Functional annotation

Differentially expressed genes were analyzed according to predefined pathways or functional categories annotated by KEGG [11], BioCarta [12], and GO [10] using the DAVID bioinformatic resource [29]. For an overrepresented GO, Biocarta or KEGG pathway, a cut-off *p *value of 0.1 was chosen. In general, it should be noted that one gene can participate in more than one KEGG or BioCarta pathway or GO category.

### Quantitative real-time PCR

Validation of the Affymetrix data was performed by qPCR analysis with the ABI Prism 7900 HT Sequence Detection System (Applied Biosystems, Foster City, CA, USA). RNA (1 μg) from BMP2 ES cells, seven-day-old control EBs and BMP2^+ ^cells were reverse transcribed with ThermoScript™ Reverse Transcriptase (Invitrogen). Then, qPCR was perfomed in triplicate for every sample using TaqMan Gene Expression Assays (Applied Biosystems). The Gene Expression Assays used for validation were Brachyury (T) (Mm00436877_m1), NF-H (Mm01191456_m1), BMP2 (Mm01340178_m1), GAPDH (Mm99999915_g1), and Nanog (Mm02019550_s1). Averaged C_t _values of each qPCR reaction from the target gene were normalized with the average C_t _values of the housekeeping gene, GAPDH, which ran in the same reaction plate, to obtain the ΔC_t _value. The fold change was calculated as follows: fold change = $2^{-(\Delta {\text{C}}_{\text{t}}\text{gene}1-\Delta {\text{C}}_{\text{t}}\text{gene}2)}$. Since the genes included are not expressed in at least one of the three conditions (BMP2 ES cells, seven-day-old control EBs or BMP2^+ ^cells), the ΔC_t _of the gene in the sample with the lowest expression was used as ΔCt gene2 to calculate the fold change using the above formula. The resulting fold change is expressed as the percentage of the maximum fold change.

## Abbreviations

AFP, α-feto protein; BMP, bone morphogenetic protein; CMV, cytomegalovirus; EB, embryoid body; ECM, extracellular matrix; EGFP, enhanced green fluorescent protein; ES, embryonic stem; FACS, fluorescence activated cell sorting; FGF, fibroblast growth factor; GFAP, glial fibrillary acidic protein; GO, Gene Ontology; KEGG, Kyoto Encyclopedia of Genes and Genomes; LIF, leukemia inhibitory factor; MAPK, mitogen activated protein kinase; NCSC, neural crest stem cell; NF, neurofilament; qPCR, quantitative real time PCR; Shh, Sonic Hedgehog; SMA, smooth muscle α actin; TGF, transforming growth factor.

## Authors' contributions

AS, MXD and JH designed the study. MXD and RHA performed the experiments. SB, MO and CW did immunohistochemical analysis. SC, JW, HS, IS, OH and NH did the Affymetrix analysis. MXD, JW, SC and AS wrote the manuscript. NGC provided the *BMP2 *promoter for this study.

## Additional data files

The following additional data are available with the online version of this manuscript: Additional data file 1 contains the RT-PCR analysis of differentiating EBs derived from the BMP2 ES cells. Additional data file 2 shows the validation of Affymetrix expression profiling data by quantitative real-time PCR. Additional data file 3 provides a list of transcripts belonging to the GO category 'transcription' that are upregulated at least two-fold in the BMP2^+ ^cells. Additional data file 4 provides a list of transcripts belonging to the GO category 'apoptosis' that are upregulated least two-fold in the BMP2^+ ^cells. Additional data file 5 provides a list of transcripts belonging to the MAPK signaling pathway that are upregulated in the BMP2^+ ^cells as well as a schematic of the KEGG MAP kinase signaling pathway indicating the genes upregulated in the BMP2^+ ^cells. Additional data file 6 provides a list of transcripts belonging to the GO category 'development' that are downregulated at least two-fold in the BMP2^+ ^cells compared to the control cells in the seven-day-old EBs. Additional data file 7 provides a list of transcripts belonging to the TGF-β signaling pathway that are specifically upregulated in the BMP2^+ ^cells as well as a schematic of the KEGG TGF-β signaling pathway indicating the upregulated genes. Additional data file 8 provides a list of transcripts belonging to the GO category 'M phase' that are specifically upregulated in the BMP2^+ ^cells. Additional data file 9 shows selected GO annotations and clustering results of genes differentially expressed in BMP2^+ ^cells. Additional data file 10 provides a video clip of β-actin CGR8 cardiomyocytes. Additional data files 11 and 12 provide video clips of BMP2^+ ^cell-derived cardiomyocytes without and with green filter, respectively. Additional data file 13 shows the immunohistochemistry results for the positive controls. Additional data file 14 contains the primers used for RT-PCR analysis. Additional data file 15 provides the normalized Affymetrix dataset of all experimental conditions, representing three independent experiments. Additional data file 16 provides the normalized Affymetrix data set for the probe sets identified to be differentially downregulated in BMP2^+ ^cells compared to control EBs. Additional data file 17 provides the normalized Affymetrix dataset for the probe sets identified to be differentially downregulated in BMP2^+ ^cells compared to control EBs and compared to undifferentiated BMP2 ES cells. Additional data file 18 provides the normalized Affymetrix dataset for the probe sets identified to be differentially upregulated in BMP2^+ ^cells compared to control EBs Additional data file 19 provides the normalized Affymetrix dataset for the probe sets identified to be differentially upregulated in BMP2^+ ^cells compared to control EBs and compared to undifferentiated BMP2 ES cells.

**Table 6:** Functional annotations (GO, KEGG, Biocarta) enriched in transcripts downregulated* in BMP2^+ ^cells compared to control cells in seven-day-old EBs and to undifferentiated BMP2 ES cells

Category	Term	Count	*p *value
GOTERM_BP_5	DNA metabolism	15	8.4E-5
GOTERM_BP_5	M phase of mitotic cell cycle	7	5.2E-4
GOTERM_BP_5	Carboxylic acid metabolism	11	2.8E-3
GOTERM_CC_5	Chromosome	9	3.8E-3
GOTERM_BP_5	Biopolymer modification	22	9.4E-3
GOTERM_MF_5	Pyrophosphatase activity	10	9.5E-3
GOTERM_BP_5	Amino acid metabolism	6	3.2E-2
GOTERM_BP_5	Recombinational repair	2	3.9E-2
GOTERM_BP_5	Cellular protein metabolism	31	4.2E-2
GOTERM_CC_5	Heterotrimeric G-protein complex	3	4.3E-2
GOTERM_MF_5	Metalloendopeptidase activity	4	5.0E-2
GOTERM_CC_5	Chromosome, periventric region	3	5.9E-2
GOTERM_CC_5	Condensed chromosome	3	6.4E-2
GOTERM_BP_5	Lipid biosynthesis	5	7.5E-2
KEGG_PATHWAY	Pyruvate metabolism	3	7.7E-2
GOTERM_BP_5	Steroid biosynthesis	3	8.3E-2
GOTERM_BP_5	Double-strand break repair	2	8.4E-2
GOTERM_BP_5	Regulation of smooth muscle contraction	2	8.4E-2
GOTERM_MF_5	Phosphoric hydrolase activity	3	9.4E-2
GOTERM_MF_5	AMP binding	2	9.7E-2

*Fold change >2, Student's *t*-test *p *value < 0.01. Count indicates the number of genes in the functional annotation category. The *p *value is from gene enrichment in annotation terms calculated by the Fisher's exact *t*-test.

## Supplementary Material

Additional data file 1 EBs were generated using the conventional hanging drop protocol (see Materials and methods and Figure 5b) and the expression of the *T-bra*, *flk1*, *smooth muscle α-actin*, *cardiac Troponin T *(*c-Troponin T*), *NF-H*, *NF-M *and *AFP *was detected using RT-PCR (for the conditions, see Additional data file 14). Click here for file

Additional data file 2 The fold change of the expression of the genes was calculated by using the formula: fold-change = $2^{-(\Delta C_{\text{t}}\text{gene}1-\Delta C_{\text{t}}\text{gene}2)}$. The Δ*C*_t _of a gene in the sample in which it is expressed at the lowest level is taken as Δ*C*_t _gene2 to calculate the fold change using the above formula. The resulting fold change is expressed as the percentage of the maximum fold change (100%) for that particular gene in every assay. Values are expressed as mean ± standard deviation (*n *= 3, technical replicates). Click here for file

Additional data file 3 Transcripts belonging to the GO category 'transcription' that are upregulated at least two-fold (*t*-test *p *value < 0.01) in the BMP2^+ ^cells compared to the control cells in the seven-day-old EBs, and those upregulated compared to the control cells in the seven-day-old EBs and the undifferentiated BMP2 ES cells. Click here for file

Additional data file 4 Transcripts belonging to the GO category 'apoptosis' that are upregulated at least two-fold (*t*-test *p *value < 0.01) in the BMP2^+ ^cells compared to the control cells in the seven-day-old EBs, and those upregulated compared to the control cells in the seven-day-old EBs and the undifferentiated BMP2 ES cells. Click here for file

Additional data file 5 Transcripts belonging to the MAPK signaling pathway that are upregulated at least two-fold (*t*-test *p *value < 0.01) in the BMP2^+ ^cells compared to the control cells in the seven-day-old EBs, and those upregulated compared to the control cells in the seven-day-old EBs and the undifferentiated BMP2 ES cells. The schematic is of the KEGG MAP kinase signaling pathway, indicating the upregulated genes (labelled with red background) in the BMP2^+ ^cells compared to the control cells in the seven-day-old EBs. Click here for file

Additional data file 6 Transcripts belonging to the GO category 'development' that are downregulated at least two-fold (*t*-test *p *value < 0.01) in the BMP2^+ ^cells compared to the control cells in the seven-day-old EBs. Click here for file

Additional data file 7 Transcripts belonging to the TGFβ signaling pathway that are specifically upregulated at least two-fold (*t*-test *p *value < 0.01) in the BMP2^+ ^cells compared to the control cells in the seven-day-old EBs as well as a schematic of the KEGG TGFβ signaling pathway indicating the upregulated genes (labelled with red background and white letters). Click here for file

Additional data file 8 Transcripts belonging to the GO category 'M phase' that are specifically upregulated at least two-fold (*t*-test *p *value < 0.01) in the BMP2^+ ^cells compared to the control cells in the seven-day-old EBs and to the undifferentiated BMP2 ES cells. Click here for file

Additional data file 9 (A,B) Pairwise comparison (BMP2 versus control EBs): 2,258 probeset IDs that were differentially expressed in BMP2^+ ^cells compared to control EBs (two-condition comparison) were converted to GenBank accession numbers and redundancies were removed (1,833 unique transcripts). Stanford Source was used to obtain GO biological process (BP) annotations. Genesis 1.7.0 was used to visualize and identify GO BP categories of interest and extract corresponding lists of transcripts. For the categories adhesion, cell cycle, cell death, cell-cell signaling, cellular metabolism, development, stress response, signal transduction, transcription and transport, 1,541 annotations were established for 1,172 transcripts. The pie chart (A) shows the distribution of these annotations. The bar chart (B) shows the number of genes in the categories adhesion, cell cycle, cell death, cell-cell signaling, cellular metabolism, development, stress response, signal transduction, transcription and transport separately for up- and downregulated transcripts. (C,D) Three-condition comparison (BMP2 versus control EBs and BMP2 ES cells). For the three-condition comparison, 551 unique transcripts were obtained from 672 probeset IDs that were differentially expressed in BMP2 versus control EBs and versus undifferentiated BMP2 ES cells using the approach described above. For the categories adhesion, cell cycle, cell death, cell-cell signaling, cellular metabolism, development, stress response, signal transduction, transcription and transport, 430 annotations were established for 268 transcripts. The pie chart (C) shows the distribution of these annotations. The bar chart (D) shows the number of genes in the categories adhesion, cell cycle, cell death, cell-cell signaling, cellular metabolism, development, stress response, signal transduction, transcription and transport separately for up- and downregulated transcripts. (E) Clustering analysis of the probesets identified as differentially expressed in the two-condition comparison (see A) and assigned to the GO category 'development'. Expression data were normalized using the RMA algorithm and hierarchical clustering was done using Cluster 2.11 as described. Visualization of the hierarchical clustering of probe sets identified as differentially expressed in the pairwise comparison (see A) and assigned to the GO category 'development'. Each probe set is represented by a single column of colored boxes; each array is represented by a single row. Cells with unchanged probe sets are colored black, upregulation is indicated with reds of increasing intensity, and downregulation with greens of increasing intensity. The dendrogram on the top of the figure represents the similarity matrix of probe sets, the dendrogram to the right the similarity matrix of arrays. Click here for file

Additional data file 10 A video clip of β-actin CGR8 cardiomyocytes. Click here for file

Additional data file 11 A video clip of BMP2^+ ^cell-derived cardiomyocytes without green filter. Click here for file

Additional data file 12 A video clip of BMP2^+ ^cell-derived cardiomyocytes with green filter. Click here for file

Additional data file 13 (A) Anti-SMA staining on mouse liver tissue. (B) MyoD1 on mouse embryonic sections. (C) α-Actinin on embryonic cardiac tissue of mouse. (D) F4/80 staining on mouse skin sections. (E) Pan cytokeratin on small intestine of mouse. (F) Ksp-Cadherin on mouse kidney sections. (G) GFAP on rat brain sections. (H) E-cadherin on mouse kidney sections. (I) Alizarin Red staining on human limb bone sections. (H) Sudan Red stainings on cytospin leukocytes. Click here for file

Additional data file 14 Primers used for RT-PCR analysis. Click here for file

Additional data file 15 Complete Affymetrix dataset for all experimental conditions, representing three independent experiments. Data were RMA-normalized as described in Materials and methods. For each probe set, the average expression value and standard deviations are given (columns B-G) as well as the change fold between conditions (columns H-J) and the results of Student's *t*-test between the experimental conditions (K-M). Expression values for the individual experimental replicates are provided in columns N-V. Click here for file

Additional data file 16 Normalized Affymetrix data set for the probe sets identified to be differentially downregulated in BMP2^+ ^cells compared to control EBs (higher than two-fold downregulation, *t*-test *p *value < 0.01). Click here for file

Additional data file 17 Normalized Affymetrix dataset for the probe sets identified to be differentially downregulated in BMP2^+ ^cells compared to control EBs and undifferentiated BMP2 ES cells (intersection of downregulation in BMP2^+ ^cells compared to control EBs (higher than two-fold downregulation, *t*-test *p *value < 0.01) and compared to undifferentiated BMP2 ES cells (higher than two-fold downregulation, *t*-test *p *value < 0.01)). Click here for file

Additional data file 18 Normalized Affymetrix dataset for the probe sets identified to be differentially upregulated in BMP2^+ ^cells compared to control EBs (higher than two-fold upregulation, *t*-test *p *value < 0.01). Click here for file

Additional data file 19 Normalized Affymetrix dataset for the probe sets identified to be differentially upregulated in BMP2^+ ^cells compared to control EBs and compared to undifferentiated BMP2 ES cells (intersection of upregulation in BMP2^+ ^cells compared to control EBs (higher than two-fold upregulation, *t*-test *p *value < 0.01) and compared to undifferentiated BMP2 ES cells (higher than two-fold upregulation, *t*-test *p *value < 0.01)). Click here for file
