# Supplementary figures and images for: Transcriptomic and phenotypic analysis of murine embryonic stem cell derived BMP2+ lineage cells: an insight into mesodermal patterning
Source: Genome Biol. 2007 Sep 4;8(9):R184. doi: 10.1186/gb-2007-8-9-r184 (PMC2375022; doi:10.1186/gb-2007-8-9-r184)

## RT-PCR analysis of differentiating EBs derived from BMP-2 ES cells

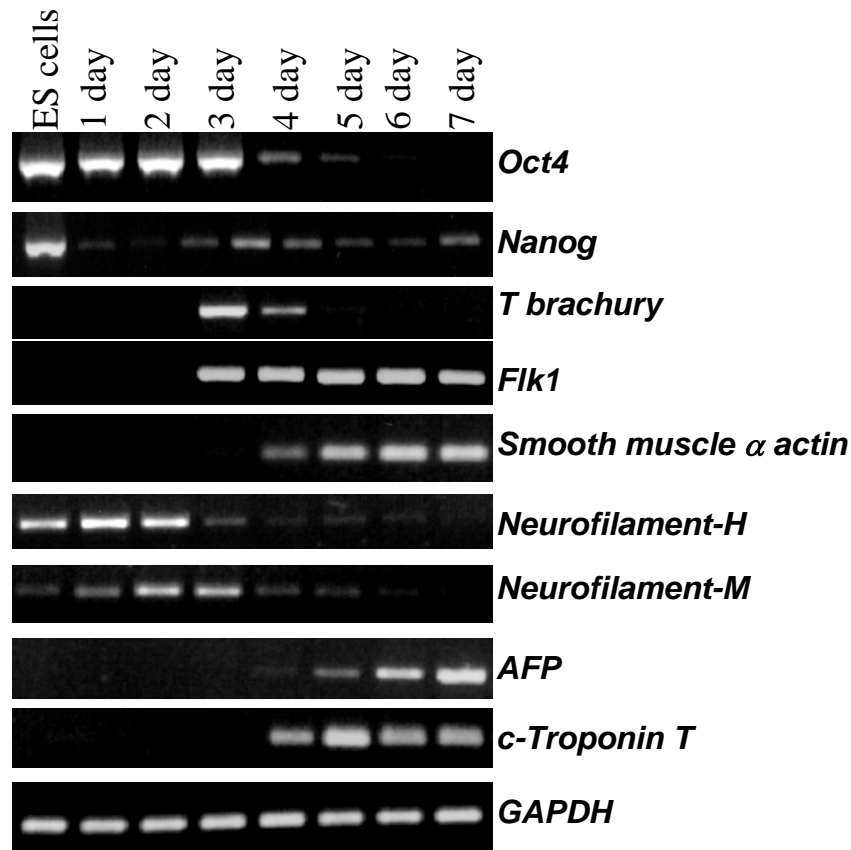

Additional data file 1

Supplement: Additional data file 1 — EBs were generated using the conventional hanging drop protocol (see Materials and methods and Figure 5b) and the expression of the T-bra, flk1, smooth muscle α-actin, cardiac Troponin T (c-Troponin T), NF-H, NF-M and AFP was detected using RT-PCR (for the conditions, see Additional data file 14). [file gb-2007-8-9-r184-S1.pdf]

## BMP-2

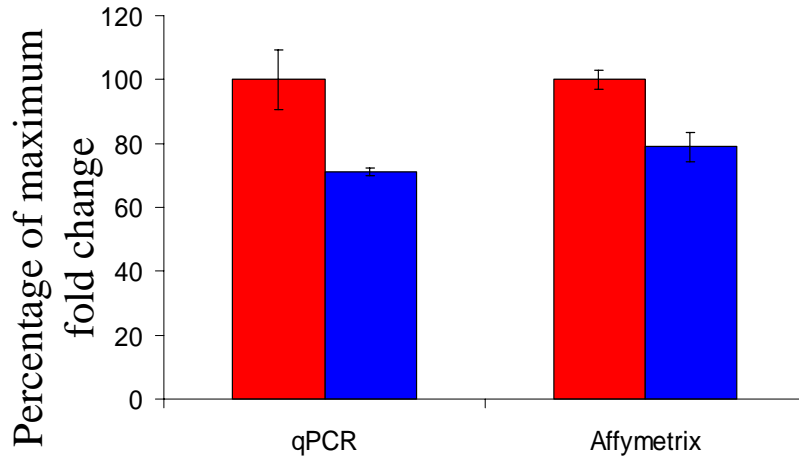

## NF-H

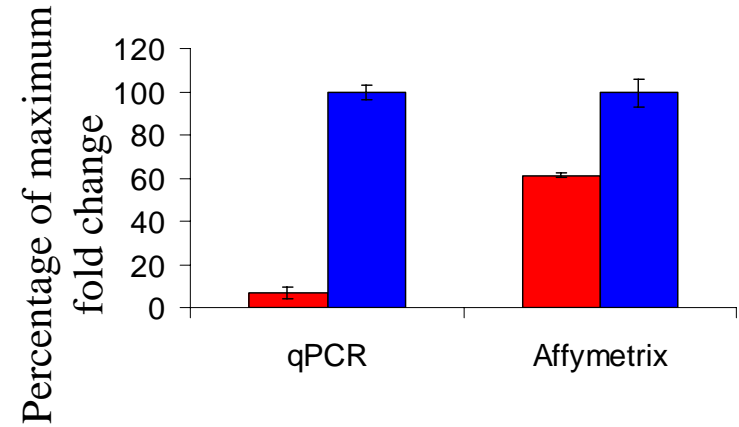

## T brachyury

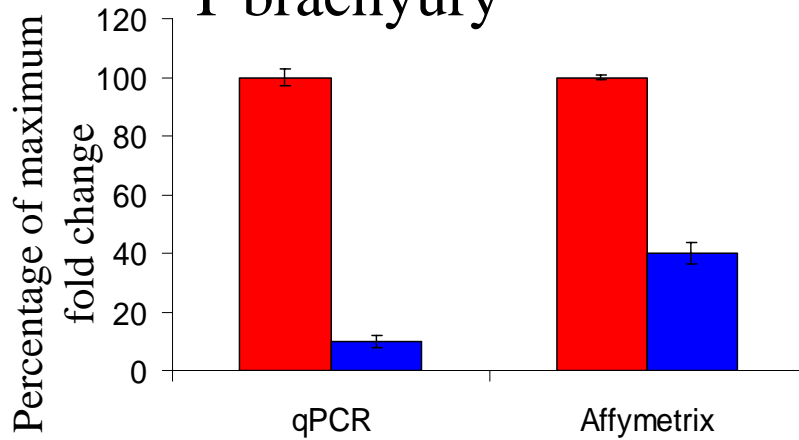

## Nanog

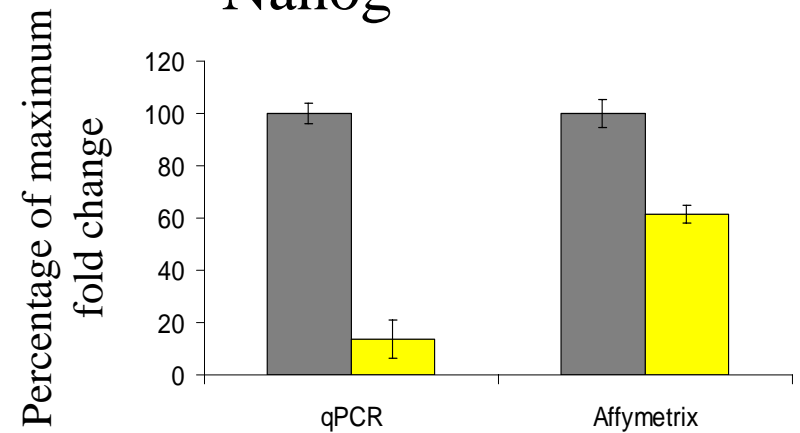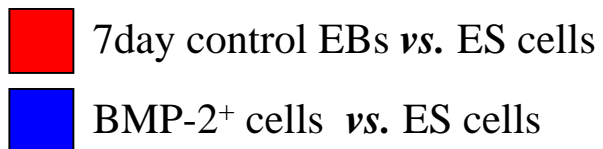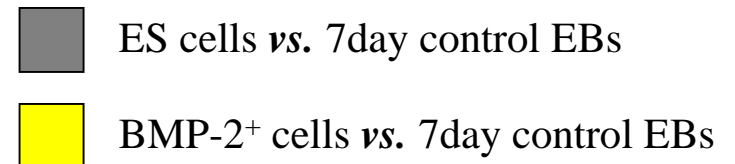

Supplement: Additional data file 2 — The fold change of the expression of the genes was calculated by using the formula: fold-change = 2−(ΔCtgene1−ΔCtgene2). The ΔCt of a gene in the sample in which it is expressed at the lowest level is taken as ΔCt gene2 to calculate the fold change using the above formula. The resulting fold change is expressed as the percentage of the maximum fold change (100%) for that particular gene in every assay. Values are expressed as mean ± standard deviation (n = 3, technical replicates). [file gb-2007-8-9-r184-S2.pdf]

**A**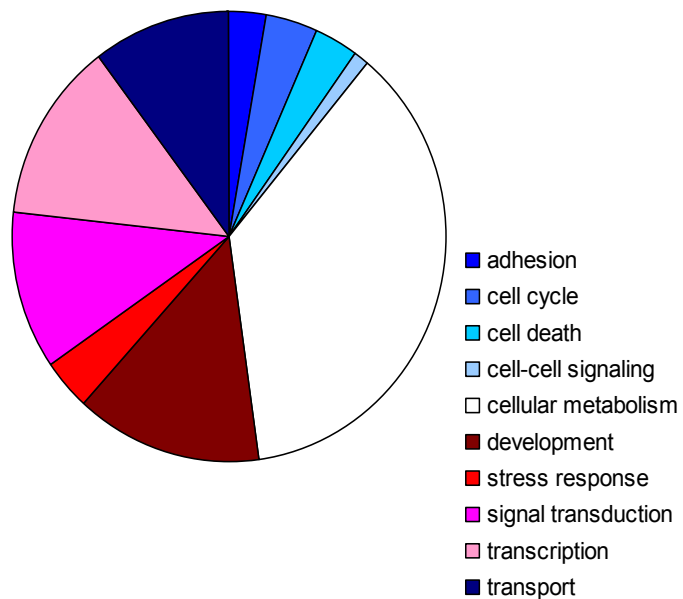**B**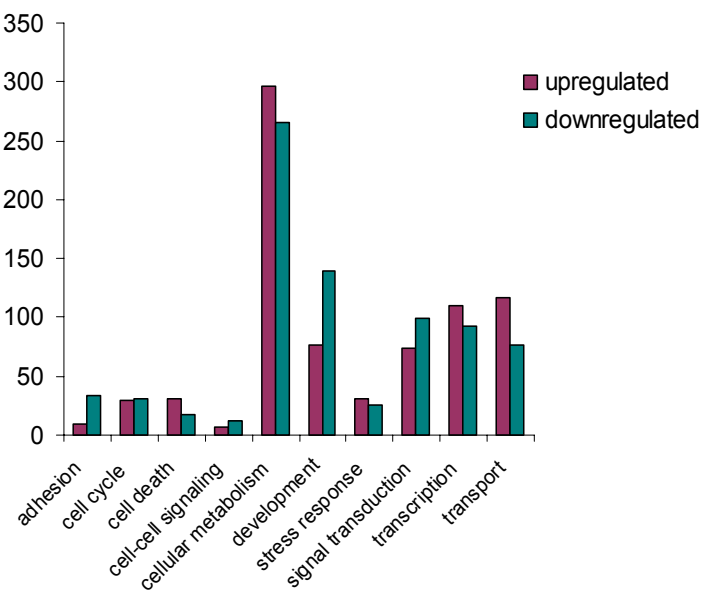**C**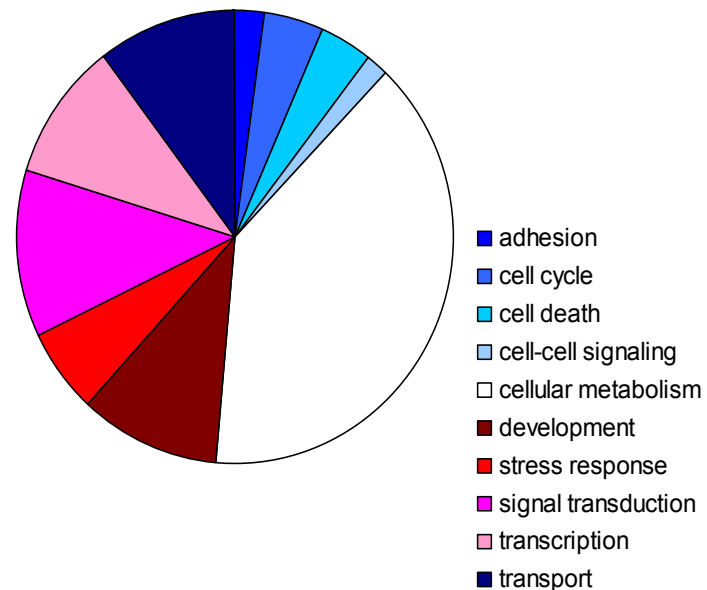**D**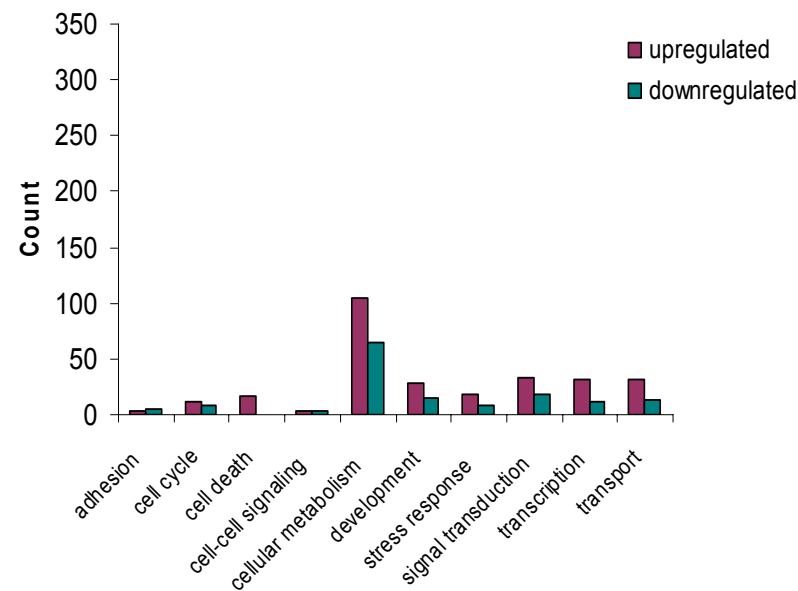

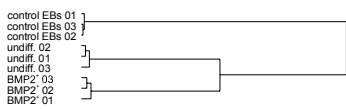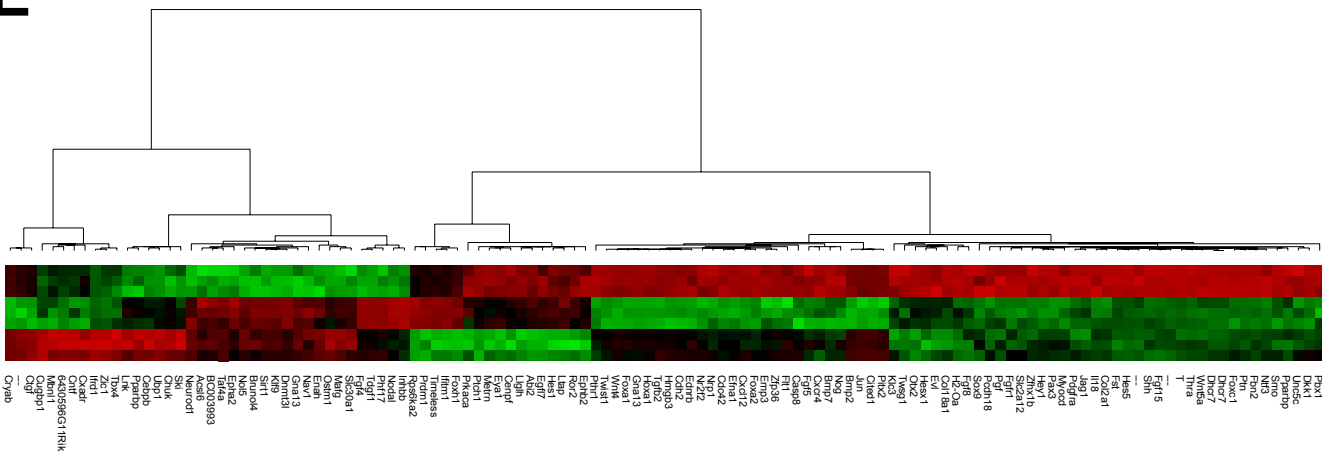

E

Supplement: Additional data file 9 — (A,B) Pairwise comparison (BMP2 versus control EBs): 2,258 probeset IDs that were differentially expressed in BMP2+ cells compared to control EBs (two-condition comparison) were converted to GenBank accession numbers and redundancies were removed (1,833 unique transcripts). Stanford Source was used to obtain GO biological process (BP) annotations. Genesis 1.7.0 was used to visualize and identify GO BP categories of interest and extract corresponding lists of transcripts. For the categories adhesion, cell cycle, cell death, cell-cell signaling, cellular metabolism, development, stress response, signal transduction, transcription and transport, 1,541 annotations were established for 1,172 transcripts. The pie chart (A) shows the distribution of these annotations. The bar chart (B) shows the number of genes in the categories adhesion, cell cycle, cell death, cell-cell signaling, cellular metabolism, development, stress response, signal transduction, transcription and transport separately for up- and downregulated transcripts. (C,D) Three-condition comparison (BMP2 versus control EBs and BMP2 ES cells). For the three-condition comparison, 551 unique transcripts were obtained from 672 probeset IDs that were differentially expressed in BMP2 versus control EBs and versus undifferentiated BMP2 ES cells using the approach described above. For the categories adhesion, cell cycle, cell death, cell-cell signaling, cellular metabolism, development, stress response, signal transduction, transcription and transport, 430 annotations were established for 268 transcripts. The pie chart (C) shows the distribution of these annotations. The bar chart (D) shows the number of genes in the categories adhesion, cell cycle, cell death, cell-cell signaling, cellular metabolism, development, stress response, signal transduction, transcription and transport separately for up- and downregulated transcripts. (E) Clustering analysis of the probesets identified as differentially expressed in the tw [file gb-2007-8-9-r184-S9.pdf]

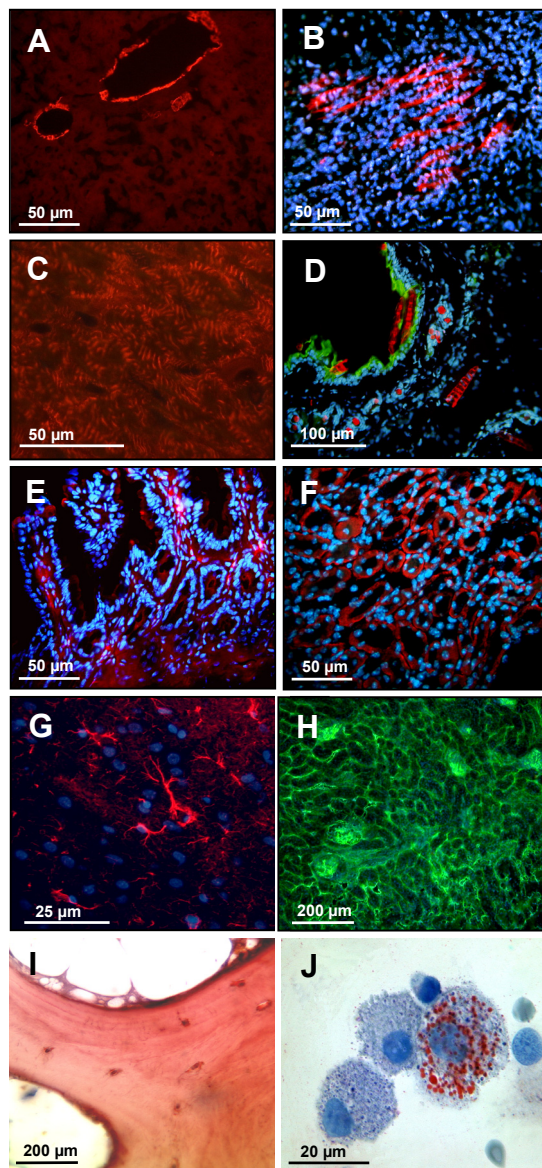

Supplement: Additional data file 13 — (A) Anti-SMA staining on mouse liver tissue. (B) MyoD1 on mouse embryonic sections. (C) α-Actinin on embryonic cardiac tissue of mouse. (D) F4/80 staining on mouse skin sections. (E) Pan cytokeratin on small intestine of mouse. (F) Ksp-Cadherin on mouse kidney sections. (G) GFAP on rat brain sections. (H) E-cadherin on mouse kidney sections. (I) Alizarin Red staining on human limb bone sections. (H) Sudan Red stainings on cytospin leukocytes. [file gb-2007-8-9-r184-S13.pdf]
