# Supplementary material for: Transcriptomic and phenotypic analysis of murine embryonic stem cell derived BMP2+ lineage cells: an insight into mesodermal patterning
Source: Genome Biol. 2007 Sep 4;8(9):R184. doi: 10.1186/gb-2007-8-9-r184 (PMC2375022; doi:10.1186/gb-2007-8-9-r184)
Supplement: Additional data file 3 — Transcripts belonging to the GO category 'transcription' that are upregulated at least two-fold (t-test p value < 0.01) in the BMP2+ cells compared to the control cells in the seven-day-old EBs, and those upregulated compared to the control cells in the seven-day-old EBs and the undifferentiated BMP2 ES cells. [file gb-2007-8-9-r184-S3.doc]

#### Additional data file 3.

#### Genes belonging to the GO category “Transcription” that are upregulated at least 2-fold (ttest<0.01) in the BMP2^+^ cells. A) compared to the control cells in the 7 days old EBs. B) compared to the control cells in the 7 days old EBs and to the undifferentiated BMP2 ES cells.

#### A

| Affymetrix ID | Gene Name | Fold Change BMP2^+^  *vs.* BMP2 7d EBs |
| --- | --- | --- |
| 1417760_at | nuclear receptor subfamily 0, group b, member 1 | 20.2 |
| 1418362_at | zinc finger protein 42 | 15.4 |
| 1417516_at | dna-damage inducible transcript 3 | 12.5 |
| 1436926_at | estrogen receptor related 2 | 7.2 |
| 1416958_at | nuclear receptor subfamily 1, group d, member 2 | 7.0 |
| 1456033_at | t-box 4 | 6.8 |
| 1456225_x_at | induced in fatty liver dystrophy 2 | 6.5 |
| 1460303_at | nuclear receptor subfamily 3, group c, member 1 | 6.3 |
| 1426412_at | neurogenic differentiation 1 | 5.7 |
| 1434681_at | retinoblastoma binding protein 7 | 5.4 |
| 1428640_at | heat shock transcription factor 2 binding protein | 4.7 |
| 1416751_a_at | dead (asp-glu-ala-asp) box polypeptide 20 | 4.7 |
| 1435302_at | taf4b rna polymerase ii, tata box binding protein (tbp)-associated factor | 4.6 |
| 1420640_at | junction-mediating and regulatory protein | 4.6 |
| 1435444_at | activating transcription factor 6 | 4.5 |
| 1418640_at | sir2 alpha | 4.3 |
| 1456341_a_at | kruppel-like factor 9 | 4.1 |
| 1456405_at | death inducer-obliterator 1 | 4.0 |
| 1449037_at | camp responsive element modulator | 4.0 |
| 1416899_at | undifferentiated embryonic cell transcription factor 1 | 4.0 |
| 1450481_at | myeloblastosis oncogene-like 1 | 4.0 |
| 1420410_at | nuclear receptor subfamily 5, group a, member 2 | 3.9 |
| 1459048_s_at | zinc finger protein 142 | 3.8 |
| 1422018_at | human immunodeficiency virus type i enhancer binding protein 2 | 3.8 |
| 1453169_a_at | general transcription factor ii h, polypeptide 1 | 3.8 |
| 1437236_a_at | zinc finger protein 110 | 3.7 |
| 1418968_at | rb1-inducible coiled-coil 1 | 3.6 |
| 1428979_at | metal response element binding transcription factor 1 | 3.6 |
| 1434882_at | metadherin | 3.6 |
| 1447864_s_at | pogo transposable element with krab domain | 3.4 |
| 1422893_at | scm-like with four mbt domains 1 | 3.4 |
| 1438725_at | thyroid hormone receptor associated protein 1 | 3.2 |
| 1427322_at | bromodomain and wd repeat domain containing 1 | 3.2 |
| 1437202_at | riken cdna c530050h10 gene | 3.2 |
| 1417945_at | pou domain, class 5, transcription factor 1 | 3.2 |
| 1417856_at | avian reticuloendotheliosis viral (v-rel) oncogene related b | 3.1 |
| 1428999_at | riken cdna 2310061n19 gene | 3.1 |
| 1421021_at | ae binding protein 2 | 3.1 |
| 1427017_at | special at-rich sequence binding protein 2 | 3.1 |
| 1434353_at | riken cdna d330030p06 gene | 3.0 |
| 1452070_at | death effector domain-containing dna binding protein 2 | 3.0 |
| 1449311_at | btb and cnc homology 1 | 3.0 |
| 1448994_at | trans-acting transcription factor 1 | 3.0 |
| 1442404_at | nucleolin | 3.0 |
| 1423169_at | taf7 rna polymerase ii, tata box binding protein (tbp)-associated factor | 3.0 |
| 1419191_at | homeodomain interacting protein kinase 3 | 2.9 |
| 1423483_s_at | tata box binding protein (tbp)-associated factor, rna polymerase i, c | 2.9 |
| 1450665_at | ga repeat binding protein, alpha | 2.9 |
| 1423582_at | doublesex and mab-3 related transcription factor 1 | 2.9 |
| 1418659_at | circadian locomoter output cycles kaput | 2.9 |
| 1436932_at | grainyhead-like 3 (drosophila) | 2.9 |
| 1436983_at | creb binding protein | 2.9 |
| 1452377_at | myeloid/lymphoid or mixed-lineage leukemia | 2.8 |
| 1418901_at | ccaat/enhancer binding protein (c/ebp), beta | 2.8 |
| 1433811_at | myeloid/lymphoid or mixed lineage-leukemia translocation to 6 homolog (drosophila) | 2.8 |
| 1429940_at | vcell division cycle 73, paf1/rna polymerase ii complex component, homolog (s. cerevisiae) | 2.8 |
| 1419867_a_at | riken cdna 4933432b13 gene | 2.7 |
| 1433633_at | interferon regulatory factor 2 binding protein 2 | 2.7 |
| 1423508_at | myst histone acetyltransferase monocytic leukemia 4 | 2.7 |
| 1424942_a_at | myelocytomatosis oncogene | 2.7 |
| 1436684_a_at | rio kinase 2 (yeast) | 2.7 |
| 1448435_at | positive cofactor 2, multiprotein complex, glutamine/q-rich-associated protein | 2.6 |
| 1428985_at | phd finger protein 22 | 2.6 |
| 1449439_at | kruppel-like factor 7 (ubiquitous) | 2.6 |
| 1456415_at | riken cdna 4930515k21 gene | 2.6 |
| 1434009_at | glucocorticoid receptor dna binding factor 1 | 2.6 |
| 1452887_at | tnf receptor-associated factor 3 interacting protein 1 | 2.6 |
| 1449592_at | transcription factor 15 | 2.6 |
| 1434054_at | v-maf musculoaponeurotic fibrosarcoma oncogene family, protein g (avian) | 2.5 |
| 1424238_at | sirtuin 7 (silent mating type information regulation 2, homolog) 7 (s. cerevisiae) | 2.5 |
| 1450072_at | ash1 (absent, small, or homeotic)-like (drosophila) | 2.5 |
| 1456103_at | promyelocytic leukemia | 2.5 |
| 1427559_a_at | activating transcription factor 2 | 2.5 |
| 1451639_at | ccaat/enhancer binding protein (c/ebp), gamma | 2.5 |
| 1422033_a_at | ciliary neurotrophic factor | 2.5 |
| 1460643_at | elongation factor rna polymerase ii | 2.5 |
| 1424407_s_at | dna segment, chr 15, brigham & women's genetics 0580 expressed | 2.4 |
| 1419295_at | camp responsive element binding protein 3-like 1 | 2.4 |
| 1440870_at | pr domain containing 16 | 2.4 |
| 1450853_at | transducin-like enhancer of split 4, homolog of drosophila e(spl) | 2.4 |
| 1436645_a_at | ccr4-not transcription complex, subunit 4 | 2.4 |
| 1435900_at | zinc finger protein 297b | 2.4 |
| 1428616_at | zinc finger protein 131 | 2.4 |
| 1460402_at | bromodomain and phd finger containing, 1 | 2.4 |
| 1418637_at | ets variant gene 3 | 2.4 |
| 1426242_at | polymerase (rna) ii (dna directed) polypeptide a | 2.4 |
| 1428688_at | programmed cell death protein 11 | 2.4 |
| 1418025_at | basic helix-loop-helix domain containing, class b2 | 2.4 |
| 1425927_a_at | transcription factor-like protein oda-10 | 2.3 |
| 1429193_at | ankyrin repeat and ibr domain containing 1 | 2.3 |
| 1420397_a_at | spen homolog, transcriptional regulator (drosophila) | 2.3 |
| 1448708_at | peroxisome proliferator activated receptor binding protein | 2.3 |
| 1417968_a_at | methyl-cpg binding domain protein 1 | 2.3 |
| 1453105_at | zinc finger protein 263 | 2.3 |
| 1449530_at | trichorhinophalangeal syndrome i (human) | 2.3 |
| 1454120_a_at | polycomb group ring finger 6 | 2.3 |
| 1419313_at | cyclin t1 | 2.3 |
| 1451814_a_at | hiv-1 tat interactive protein 2, homolog (human) | 2.2 |
| 1434179_at | myeloid/lymphoid or mixed-lineage leukemia 3 | 2.2 |
| 1429128_x_at | nuclear factor of kappa light polypeptide gene enhancer in b-cells 2, p49/p100 | 2.2 |
| 1427408_a_at | thyroid hormone receptor associated protein 3 | 2.2 |
| 1416543_at | nuclear factor, erythroid derived 2, like 2 | 2.2 |
| 1434284_at | riken cdna g630013p12 gene | 2.2 |
| 1452438_s_at | taf4a rna polymerase ii, tata box binding protein (tbp)-associated factor | 2.2 |
| 1437479_x_at | t-box 3 | 2.2 |
| 1454938_at | sorting nexin 13 | 2.2 |
| 1428232_at | cleavage and polyadenylation specific factor 6 | 2.2 |
| 1419641_at | purine rich element binding protein b | 2.2 |
| 1419536_a_at | v-rel reticuloendotheliosis viral oncogene homolog a (avian) | 2.2 |
| 1420497_a_at | ccaat/enhancer binding protein zeta | 2.2 |
| 1454900_s_at | pam, highwire, rpm 1 | 2.1 |
| 1417791_a_at | zinc finger, matrin-like | 2.1 |
| 1453760_at | mesoderm induction early response 1 homolog (xenopus laevis | 2.1 |
| 1455323_at | rb-associated krab repressor | 2.1 |
| 1451569_at | nuclear receptor subfamily 2, group c, member 2 | 2.1 |
| 1434037_s_at | p300/cbp-associated factor | 2.1 |
| 1416019_at | down-regulator of transcription 1 | 2.1 |
| 1437302_at | adrenergic receptor, beta 2 | 2.1 |
| 1423430_at | myb binding protein (p160) 1a | 2.1 |
| 1455121_at | riken cdna a630025c20 gene | 2.1 |
| 1418301_at | interferon regulatory factor 6 | 2.1 |
| 1426827_at | riken cdna a730098d12 gene | 2.0 |
| 1418801_at | zinc finger with krab and scan domains 1 | 2.0 |
| 1444779_s_at | zinc finger protein 59 | 2.0 |
| 1450050_at | histone cell cycle regulation defective homolog a (s. cerevisiae) | 2.0 |
| 1418978_at | sap30 binding protein | 2.0 |
| 1433557_at | chromobox homolog 7 | 2.0 |

**B**

| Affymetrix ID | Gene Name | Fold Change BMP2^+^  *vs.* BMP2 7d EBs | **Fold Change**  BMP2^+^  *vs.* BMP2 ES |
| --- | --- | --- | --- |
| 1457033_at | gene model 397, (NCBI); Mus musculus sequence XM_486399; similar to Gm397 protein | 41.4 | 12.8 |
| 1456033_at | T-box 4 | 6.8 | 6.4 |
| 1457635_s_at | nuclear receptor subfamily 3, group C, member 1 | 4.7 | 3.0 |
| 1420640_at | junction-mediating and regulatory protein | 4.6 | 8.3 |
| 1449037_at | cAMP responsive element modulator | 4.0 | 4.4 |
| 1450481_at | --- | 4.0 | 3.7 |
| 1450957_a_at | sequestosome 1 | 3.9 | 2.7 |
| 1422018_at | human immunodeficiency virus type I enhancer binding protein 2 | 3.8 | 11.2 |
| 1453169_a_at | general transcription factor II H, polypeptide 1 | 3.8 | 2.4 |
| 1459048_s_at | zinc finger protein 142 | 3.8 | 3.1 |
| 1434882_at | Metadherin | 3.6 | 3.4 |
| 1418968_at | RB1-inducible coiled-coil 1 | 3.6 | 3.4 |
| 1452163_at | E26 avian leukemia oncogene 1, 5' domain | 3.5 | 77.9 |
| 1422893_at | Scm-like with four mbt domains 1 | 3.4 | 3.9 |
| 1447864_s_at | pogo transposable element with KRAB domain | 3.4 | 9.8 |
| 1438725_at | thyroid hormone receptor associated protein 1 | 3.2 | 7.6 |
| 1440085_at | ectodysplasin A2 isoform receptor | 3.1 | 3.2 |
| 1428999_at | PHD finger protein 3 | 3.1 | 2.2 |
| 1434353_at | Scm-like with four mbt domains 2 | 3.0 | 10.9 |
| 1419191_at | homeodomain interacting protein kinase 3 | 2.9 | 4.2 |
| 1433633_at | interferon regulatory factor 2 binding protein 2 | 2.7 | 4.3 |
| 1436684_a_at | RIO kinase 2 (yeast) | 2.7 | 2.1 |
| 1424942_a_at | myelocytomatosis oncogene | 2.7 | 2.0 |
| 1438487_s_at | zinc finger, ZZ domain containing 3 | 2.7 | 3.5 |
| 1449439_at | Kruppel-like factor 7 (ubiquitous) | 2.6 | 7.2 |
| 1434009_at | RIKEN cDNA 6430596G11 gene | 2.6 | 3.4 |
| 1427559_a_at | activating transcription factor 2 | 2.5 | 3.3 |
| 1450072_at | ash1 (absent, small, or homeotic)-like (Drosophila) | 2.5 | 2.5 |
| 1427971_at | Vcell division cycle 73, Paf1/RNA polymerase II complex component, homolog (S, cerevisiae) | 2.5 | 2.9 |
| 1422033_a_at | ciliary neurotrophic factor | 2.5 | 3.2 |
| 1429217_at | zinc finger protein 655 | 2.5 | 2.5 |
| 1430535_at | TSC22 domain family 2 | 2.4 | 2.8 |
| 1418025_at | basic helix-loop-helix domain containing, class B2 | 2.4 | 3.7 |
| 1418637_at | ets variant gene 3 | 2.4 | 2.0 |
| 1419295_at | cAMP responsive element binding protein 3-like 1 | 2.4 | 2.5 |
| 1419641_at | purine rich element binding protein B | 2.2 | 6.0 |
| 1429128_x_at | nuclear factor of kappa light polypeptide gene enhancer in B-cells 2, p49/p100 | 2.2 | 2.2 |
| 1428232_at | cleavage and polyadenylation specific factor 6 | 2.2 | 3.2 |
| 1419536_a_at | v-rel reticuloendotheliosis viral oncogene homolog A (avian) | 2.2 | 3.1 |
| 1454938_at | sorting nexin 13 | 2.2 | 3.6 |
| 1455121_at | RIKEN cDNA A630025C20 gene | 2.1 | 3.4 |
| 1416019_at | down-regulator of transcription 1 | 2.1 | 2.3 |
| 1418301_at | interferon regulatory factor 6 | 2.1 | 4.2 |
| 1437302_at | adrenergic receptor, beta 2 | 2.1 | 3.7 |
| 1426827_at | RIKEN cDNA A730098D12 gene | 2.0 | 2.0 |
| 1438255_at | checkpoint supressor 1 | 2.0 | 3.3 |
