# Supplementary material for: Transcriptomic and phenotypic analysis of murine embryonic stem cell derived BMP2+ lineage cells: an insight into mesodermal patterning
Source: Genome Biol. 2007 Sep 4;8(9):R184. doi: 10.1186/gb-2007-8-9-r184 (PMC2375022; doi:10.1186/gb-2007-8-9-r184)
Supplement: Additional data file 4 — Transcripts belonging to the GO category 'apoptosis' that are upregulated at least two-fold (t-test p value < 0.01) in the BMP2+ cells compared to the control cells in the seven-day-old EBs, and those upregulated compared to the control cells in the seven-day-old EBs and the undifferentiated BMP2 ES cells. [file gb-2007-8-9-r184-S4.doc]

**Additional data file**

**4.** Genes belonging to the GO category “Apoptosis” that are upregulated at least 2-fold (ttest<0.01) in the BMP2^+^ cells. **A**) compared to the control cells in the 7 days old EBs. **B**) compared to the control cells in the 7 days old EBs and to the undifferentiated BMP2 ES cells.

A

| **Affymetrix ID** | Gene Name | **Change Fold**  BMP2^+^ *vs.* BMP2 7d EBs |
| --- | --- | --- |
| 1427126_at | heat shock protein 1b | 65.4 |
| 1417516_at | dna-damage inducible transcript 3 | 12.5 |
| 1420086_x_at | fibroblast growth factor 4 | 9.4 |
| 1456225_x_at | induced in fatty liver dystrophy 2 | 6.5 |
| 1418572_x_at | tumor necrosis factor receptor superfamily, member 12a | 5.6 |
| 1449254_at | secreted phosphoprotein 1 | 5.6 |
| 1420640_at | junction-mediating and regulatory protein | 4.6 |
| 1418640_at | sir2 alpha | 4.3 |
| 1456405_at | death inducer-obliterator 1 | 4.0 |
| 1450957_a_at | sequestosome 1 | 3.9 |
| 1452179_at | phd finger protein 17 | 3.6 |
| 1418110_a_at | inositol polyphosphate-5-phosphatase d | 3.5 |
| 1435040_at | interleukin-1 receptor-associated kinase 3 | 3.1 |
| 1416041_at | serum/glucocorticoid regulated kinase | 3.0 |
| 1452070_at | death effector domain-containing dna binding protein 2 | 3.0 |
| 1415893_at | sphingosine phosphate lyase 1 | 2.9 |
| 1419191_at | homeodomain interacting protein kinase 3 | 2.9 |
| 1418901_at | ccaat/enhancer binding protein (c/ebp), beta | 2.8 |
| 1416926_at | transformation related protein 53 inducible nuclear protein 1 | 2.7 |
| 1424641_a_at | tho complex 1 | 2.7 |
| 1424942_a_at | myelocytomatosis oncogene | 2.7 |
| 1418203_at | phorbol-12-myristate-13-acetate-induced protein 1 | 2.6 |
| 1416271_at | perp, tp53 apoptosis effector | 2.5 |
| 1448399_at | tax1 (human t-cell leukemia virus type i) binding protein 1 | 2.4 |
| 1435369_at | expressed sequence c78212 | 2.4 |
| 1428688_at | programmed cell death protein 11 | 2.4 |
| 1425927_a_at | transcription factor-like protein oda-10 | 2.3 |
| 1427448_at | rabaptin, rab gtpase binding effector protein 1 | 2.3 |
| 1417658_at | transforming growth factor beta regulated gene 4 | 2.3 |
| 1456080_a_at | serine incorporator 3 | 2.3 |
| 1436157_at | cell division cycle and apoptosis regulator 1 | 2.3 |
| 1452050_at | calcium/calmodulin-dependent protein kinase id | 2.2 |
| 1451814_a_at | hiv-1 tat interactive protein 2, homolog (human) | 2.2 |
| 1421679_a_at | cyclin-dependent kinase inhibitor 1a (p21) | 2.2 |
| 1422452_at | bcl2-associated athanogene 3 | 2.2 |
| 1424594_at | lectin, galactose binding, soluble 7 | 2.1 |
| 1449485_at | receptor (tnfrsf)-interacting serine-threonine kinase 1 | 2.0 |
| 1460251_at | fas (tnf receptor superfamily member) | 2.0 |

**B**

| **Affymetrix ID** | Gene Name | **Fold Change**  BMP2^+^  *vs.* BMP2 7d EBs | **Fold Change**  BMP2^+^  *vs.* BMP2 ES |
| --- | --- | --- | --- |
| 1427126_at | heat shock protein 1A | 65.4 | 124.6 |
| 1418572_x_at | tumor necrosis factor receptor superfamily, member 12a | 5.6 | 30.9 |
| 1420640_at | junction-mediating and regulatory protein | 4.6 | 8.3 |
| 1450957_a_at | sequestosome 1 | 3.9 | 2.7 |
| 1415893_at | sphingosine phosphate lyase 1 | 2.9 | 2.8 |
| 1419191_at | homeodomain interacting protein kinase 3 | 2.9 | 4.2 |
| 1424942_a_at | myelocytomatosis oncogene | 2.7 | 2.0 |
| 1416926_at | transformation related protein 53 inducible nuclear protein 1 | 2.7 | 2.6 |
| 1418203_at | phorbol-12-myristate-13-acetate-induced protein 1 | 2.6 | 3.6 |
| 1416271_at | PERP, TP53 apoptosis effector | 2.5 | 13.2 |
| 1456080_a_at | tumor differentially expressed 1 | 2.3 | 2.3 |
| 1427448_at | rabaptin, RAB GTPase binding effector protein 1 | 2.3 | 2.8 |
| 1421679_a_at | cyclin-dependent kinase inhibitor 1A (P21) | 2.2 | 4.2 |
| 1422452_at | Bcl2-associated athanogene 3 | 2.2 | 3.2 |
| 1424594_at | lectin, galactose binding, soluble 7 | 2.1 | 31.1 |
| 1449485_at | receptor (TNFRSF)-interacting serine-threonine kinase 1 | 2.0 | 2.3 |
| 1460251_at | Fas (TNF receptor superfamily member) | 2.0 | 2.8 |
