# Supplementary material for: Transcriptomic and phenotypic analysis of murine embryonic stem cell derived BMP2+ lineage cells: an insight into mesodermal patterning
Source: Genome Biol. 2007 Sep 4;8(9):R184. doi: 10.1186/gb-2007-8-9-r184 (PMC2375022; doi:10.1186/gb-2007-8-9-r184)
Supplement: Additional data file 5 — Transcripts belonging to the MAPK signaling pathway that are upregulated at least two-fold (t-test p value < 0.01) in the BMP2+ cells compared to the control cells in the seven-day-old EBs, and those upregulated compared to the control cells in the seven-day-old EBs and the undifferentiated BMP2 ES cells. The schematic is of the KEGG MAP kinase signaling pathway, indicating the upregulated genes (labelled with red background) in the BMP2+ cells compared to the control cells in the seven-day-old EBs. [file gb-2007-8-9-r184-S5.doc]

**Additional data file 5.**  Genes belonging to the MAPK Signaling pathway that are up regulated at least 2-fold (ttest<0.01) in the BMP2^+^ cells. **A**) compared to the control cells in the 7 days old EBs. **B**) compared to the control cells in the 7 day old EBs and to the undifferentiated BMP2 ES cells. **C)** Schematic of the KEGG MAP kinase signaling pathway indicating the upregulated genes (labelled with red background and white letters).

A

| Affymetrix ID | Gene Name | Fold Change BMP2^+^  *vs.* BMP2 7d EBs |
| --- | --- | --- |
| 1427126_at | heat shock protein 1A | 65.4 |
| 1417516_at | DNA-damage inducible transcript 3 | 12.5 |
| 1420086_x_at | fibroblast growth factor 4 | 9.4 |
| 1434815_a_at | mitogen-activated protein kinase-activated protein kinase 3 | 4.1 |
| 1418497_at | fibroblast growth factor 13 | 3.8 |
| 1437015_x_at | phospholipase A2, group IB, pancreas | 3.6 |
| 1431182_at | heat shock protein 8; similar to heat shock protein 8; similar to heat shock protein 8; similar to Heat shock cognate 71 kDa protein | 3.6 |
| 1449519_at | growth arrest and DNA-damage-inducible 45 alpha | 3.2 |
| 1418504_at | heat shock protein 9A | 2.9 |
| 1436015_s_at | serine/threonine kinase 4 | 2.9 |
| 1434472_at | dual specificity phosphatase 3 (vaccinia virus phosphatase VH1-related) | 2.8 |
| 1424942_a_at | myelocytomatosis oncogene | 2.7 |
| 1460608_at | calcium channel, voltage-dependent, N type, alpha 1B subunit | 2.7 |
| 1427464_s_at | heat shock 70kD protein 5 (glucose-regulated protein) | 2.7 |
| 1422943_a_at | heat shock protein 1 | 2.7 |
| 1427559_a_at | activating transcription factor 2 | 2.5 |
| 1417016_at | MAP kinase-activated protein kinase 5 | 2.5 |
| 1451383_a_at | conserved helix-loop-helix ubiquitous kinase | 2.4 |
| 1429128_x_at | nuclear factor of kappa light polypeptide gene enhancer in B-cells 2, p49/p100 | 2.2 |
| 1418401_a_at | dual specificity phosphatase 16 | 2.2 |
| 1418943_at | RIKEN cDNA B230120H23 gene | 2.1 |
| 1421877_at | mitogen activated protein kinase 9 | 2.1 |
| 1451502_at | phospholipase A2, group X | 2.1 |
| 1460251_at | Fas (TNF receptor superfamily member) | 2.0 |

B

| Affymetrix ID | Gene Name | Fold Change BMP2^+^  *vs.* BMP2 7d EBs | **Fold Change**  BMP2^+^  *vs.* BMP2 ES |
| --- | --- | --- | --- |
| 1427126_at | heat shock protein 1A | 65.4 | 124.6 |
| 1418497_at | fibroblast growth factor 13 | 3.8 | 7.0 |
| 1436015_s_at | serine/threonine kinase 4 | 2.9 | 2.2 |
| 1424942_a_at | myelocytomatosis oncogene | 2.7 | 2.0 |
| 1427464_s_at | heat shock 70kD protein 5 (glucose-regulated protein) | 2.7 | 4.3 |
| 1460608_at | calcium channel, voltage-dependent, N type, alpha 1B subunit | 2.7 | 3.1 |
| 1427559_a_at | activating transcription factor 2 | 2.5 | 3.3 |
| 1429128_x_at | nuclear factor of kappa light polypeptide gene enhancer in B-cells 2, p49/p100 | 2.2 | 2.2 |
| 1460251_at | Fas (TNF receptor superfamily member) | 2.0 | 2.8 |

**C**
