# Supplementary material for: Transcriptomic and phenotypic analysis of murine embryonic stem cell derived BMP2+ lineage cells: an insight into mesodermal patterning
Source: Genome Biol. 2007 Sep 4;8(9):R184. doi: 10.1186/gb-2007-8-9-r184 (PMC2375022; doi:10.1186/gb-2007-8-9-r184)
Supplement: Additional data file 6 — Transcripts belonging to the GO category 'development' that are downregulated at least two-fold (t-test p value < 0.01) in the BMP2+ cells compared to the control cells in the seven-day-old EBs. [file gb-2007-8-9-r184-S6.doc]

**Additional data file 6** Genes belonging to the GO category “Development” that are downregulated at least 2-fold (ttest<0.01) in the BMP2^+^ cells compared to the control cells in the 7 days old EBs.

| Affymetrix ID | Gene Name | Fold Change BMP2^+^  *vs.* BMP2 7d EBs |
| --- | --- | --- |
| 1448254_at | pleiotrophin | -39.8 |
| 1419304_at | brachyury | -30.0 |
| 1436869_at | sonic hedgehog | -13.2 |
| 1456010_x_at | hairy and enhancer of split 5 (drosophila) | -9.8 |
| 1442025_a_at | zinc finger and btb domain containing 16 | -9.8 |
| 1454830_at | fibrillin 2 | -9.1 |
| 1425926_a_at | orthodenticle homolog 2 (drosophila) | -7.8 |
| 1418376_at | fibroblast growth factor 15 | -7.1 |
| 1457424_at | eyes absent 1 homolog (drosophila) | -6.9 |
| 1434458_at | follistatin | -6.8 |
| 1428853_at | patched homolog 1 | -6.6 |
| 1419485_at | forkhead box c1 | -6.6 |
| 1415999_at | hairy/enhancer-of-split related with yrpw motif 1 | -6.0 |
| 1458232_at | dickkopf homolog 1 (xenopus laevis) | -6.0 |
| 1420604_at | homeo box gene expressed in es cells | -5.9 |
| 1421917_at | platelet derived growth factor receptor, alpha polypeptide | -5.4 |
| 1418084_at | neuropilin 1 | -5.3 |
| 1448710_at | chemokine (c-x-c motif) receptor 4 | -5.1 |
| 1448510_at | ephrin a1 | -5.1 |
| 1425978_at | myocardin | -5.1 |
| 1424254_at | interferon induced transmembrane protein 1 | -5.1 |
| 1450567_a_at | procollagen, type ii, alpha 1 | -5.1 |
| 1451882_a_at | fibroblast growth factor 8 | -4.8 |
| 1450803_at | neurotrophin 3 | -4.5 |
| 1423250_a_at | transforming growth factor, beta 2 | -4.4 |
| 1417574_at | chemokine (c-x-c motif) ligand 12 | -4.4 |
| 1420425_at | pr domain containing 1, with znf domain | -4.3 |
| 1418496_at | forkhead box a1 | -4.0 |
| 1418102_at | hairy and enhancer of split 1 (drosophila) | -4.0 |
| 1434070_at | jagged 1 | -3.8 |
| 1418910_at | bone morphogenetic protein 7 | -3.8 |
| 1425016_at | eph receptor b2 | -3.7 |
| 1426955_at | procollagen, type xviii, alpha 1 | -3.6 |
| 1418815_at | cadherin 2 | -3.6 |
| 1417092_at | parathyroid hormone receptor 1 | -3.5 |
| 1417104_at | epithelial membrane protein 3 | -3.5 |
| 1452519_a_at | zinc finger protein 36 | -3.5 |
| 1419297_at | histocompatibility 2, o region alpha locus | -3.4 |
| 1436791_at | wingless-related mmtv integration site 5a | -3.4 |
| 1423428_at | receptor tyrosine kinase-like orphan receptor 2 | -3.3 |
| 1418733_at | twist gene homolog 1 (drosophila) | -3.3 |
| 1437779_at | forkhead box h1 | -3.1 |
| 1433985_at | abl-interactor 2 | -3.1 |
| 1418471_at | placental growth factor | -3.1 |
| 1454675_at | thyroid hormone receptor alpha | -3.1 |
| 1448619_at | 7-dehydrocholesterol reductase | -3.0 |
| 1425383_a_at | pre b-cell leukemia transcription factor 1 | -3.0 |
| 1441743_at | paired box gene 3 | -3.0 |
| 1422889_at | protocadherin 18 | -2.9 |
| 1451428_x_at | egf-like domain 7 | -2.9 |
| 1424050_s_at | fibroblast growth factor receptor 1 | -2.9 |
| 1423635_at | bone morphogenetic protein 2 | -2.9 |
| 1436475_at | nuclear receptor subfamily 2, group f, member 2 | -2.8 |
| 1438883_at | fibroblast growth factor 5 | -2.8 |
| 1417932_at | interleukin 18 | -2.8 |
| 1427100_at | meteorin, glial cell differentiation regulator | -2.7 |
| 1435911_s_at | tata box binding protein-like 1 | -2.7 |
| 1427049_s_at | smoothened homolog (drosophila) | -2.7 |
| 1422748_at | zinc finger homeobox 1b | -2.7 |
| 1448694_at | jun oncogene | -2.6 |
| 1416155_at | high mobility group box 3 | -2.6 |
| 1424552_at | caspase 8 | -2.6 |
| 1451538_at | sry-box containing gene 9 | -2.5 |
| 1427161_at | riken cdna 6530404a22 gene | -2.5 |
| 1449031_at | cbp/p300-interacting transactivator with glu/asp-rich carboxy-terminal domain 1 | -2.5 |
| 1435807_at | cell division cycle 42 homolog (s. cerevisiae) | -2.5 |
| 1455592_at | loop tail associated protein | -2.3 |
| 1416621_at | lethal giant larvae homolog | -2.3 |
| 1434920_a_at | ena-vasodilator stimulated phosphoprotein | -2.3 |
| 1424797_a_at | paired-like homeodomain transcription factor 2 | -2.3 |
| 1430295_at | guanine nucleotide binding protein, alpha 13 | -2.2 |
| 1447720_x_at | protein kinase, camp dependent, catalytic, alpha | -2.2 |
| 1450782_at | wingless-related mmtv integration site 4 | -2.2 |
| 1417542_at | ribosomal protein s6 kinase, related sequence 1 | -2.2 |
| 1417586_at | timeless homolog (drosophila) | -2.2 |
| 1449522_at | unc-5 homolog c (c. elegans) | -2.2 |
| 1450388_s_at | twisted gastrulation homolog 1 (drosophila) | -2.1 |
| 1422300_at | noggin | -2.1 |
| 1437347_at | endothelin receptor type b | -2.0 |
| 1425558_at | kinesin light chain 3 | -2.0 |
| 1440926_at | fms-like tyrosine kinase 1 | -2.0 |
| 1420565_at | homeo box a1 | -2.0 |
