# Supplementary material for: Transcriptomic and phenotypic analysis of murine embryonic stem cell derived BMP2+ lineage cells: an insight into mesodermal patterning
Source: Genome Biol. 2007 Sep 4;8(9):R184. doi: 10.1186/gb-2007-8-9-r184 (PMC2375022; doi:10.1186/gb-2007-8-9-r184)
Supplement: Additional data file 7 — Transcripts belonging to the TGFβ signaling pathway that are specifically upregulated at least two-fold (t-test p value < 0.01) in the BMP2+ cells compared to the control cells in the seven-day-old EBs as well as a schematic of the KEGG TGFβ signaling pathway indicating the upregulated genes (labelled with red background and white letters). [file gb-2007-8-9-r184-S7.doc]

**Additional data file 7.** Genes belonging to the TGFβ signaling pathway that are specifically upregulated at least 2-fold (ttest<0.01) in the BMP2^+^ cells compared to the control cells in the 7 day old EBs. **A**) list of the genes. **B**) Schematic of the KEGG TGFβ signaling pathway indicating the upregulated genes (labelled with red background and white letters).

**A**

| Affymetrix ID | Gene Name | Fold Change BMP2^+^  *vs.* BMP2 7d EBs |
| --- | --- | --- |
| 1455851_at | bone morphogenetic protein 5 | -12.6 |
| 1434458_at | follistatin | -6.8 |
| 1425895_a_at | inhibitor of dna binding 1 | -4.5 |
| 1423250_a_at | transforming growth factor, beta 2 | -4.4 |
| 1448870_at | latent transforming growth factor beta binding protein 1 | -4.0 |
| 1418910_at | bone morphogenetic protein 7 | -3.8 |
| 1416623_at | thrombospondin 3 | -3.8 |
| 1425166_at | retinoblastoma-like 1 (p107) | -3.4 |
| 1423635_at | bone morphogenetic protein 2 | -2.9 |
| 1460207_s_at | e2f transcription factor 5 | -2.5 |
| 1422771_at | mad homolog 6 (drosophila) | -2.5 |
| 1423259_at | inhibitor of dna binding 4 | -2.5 |
| 1424797_a_at | paired-like homeodomain transcription factor 2 | -2.3 |
| 1422300_at | noggin | -2.1 |

## B
