# Supplementary material for: Transcriptomic and phenotypic analysis of murine embryonic stem cell derived BMP2+ lineage cells: an insight into mesodermal patterning
Source: Genome Biol. 2007 Sep 4;8(9):R184. doi: 10.1186/gb-2007-8-9-r184 (PMC2375022; doi:10.1186/gb-2007-8-9-r184)
Supplement: Additional data file 8 — Transcripts belonging to the GO category 'M phase' that are specifically upregulated at least two-fold (t-test p value < 0.01) in the BMP2+ cells compared to the control cells in the seven-day-old EBs and to the undifferentiated BMP2 ES cells. [file gb-2007-8-9-r184-S8.doc]

**Additional data file 8** Genes belonging to the GO category “M Phase” that are specifically upregulated at least 2-fold (ttest<0.01) in the BMP2^+^ cells compared to the control cells in the 7 day old EBs and to the undifferentiated BMP2 ES cells

| Affymetrix ID | Gene Name | Fold Change BMP2^+^  *vs.* BMP2 7d EBs | **Fold Change**  BMP2^+^  *vs.* BMP2 ES |
| --- | --- | --- | --- |
| 1421963_a_at | cell division cycle 25 homolog B (S. cerevisiae) | -3.2 | -2.9 |
| 1423847_at | RIKEN cDNA 2810406C15 gene | -3 | -3.3 |
| 1424046_at | budding uninhibited by benzimidazoles 1 homolog (S. cerevisiae) | -2.7 | -2.7 |
| 1439510_at | shugoshin-like 1 (S, pombe) | -2.5 | -6.1 |
| 1447363_s_at | budding uninhibited by benzimidazoles 1 homolog, beta (S. cerevisiae) | -2.4 | -3.2 |
| 1417542_at | ribosomal protein S6 kinase, polypeptide 2 | -2.2 | -4.5 |
| 1452459_at | asp (abnormal spindle)-like, microcephaly associated (Drosophila) | -2.2 | -2.8 |
