# Supplementary material for: Transcriptomic and phenotypic analysis of murine embryonic stem cell derived BMP2+ lineage cells: an insight into mesodermal patterning
Source: Genome Biol. 2007 Sep 4;8(9):R184. doi: 10.1186/gb-2007-8-9-r184 (PMC2375022; doi:10.1186/gb-2007-8-9-r184)
Supplement: Additional data file 14 — Primers used for RT-PCR analysis. [file gb-2007-8-9-r184-S14.doc]

**Additional data file 14** Sequences of the optimal primers for RT-PCR analysis of total RNA isolated from ES cells and EBs

| **Gene** | **NCBI Accession ID** | **forwardprimer** | **reverseprimer** | **PCR Fragment (bp)** |
| --- | --- | --- | --- | --- |
| activin | [AB029485.](http://www.ncbi.nlm.nih.gov/entrez/viewer.fcgi?db=nucleotide&val=5381219) | cctagaaagcgggacctatga | tacacgggtgcagggtaaggc | 311 |
| adipsin | [NM_013459.](http://www.ncbi.nlm.nih.gov/entrez/viewer.fcgi?db=nucleotide&val=7304866) | tggtatgatgtgcagagtgtagtg | ggtaggatgacactcgggtatag | 454 |
| AFP | [NM_007423](http://www.ncbi.nlm.nih.gov/entrez/viewer.fcgi?db=nucleotide&val=31982512) | ccagaacctgccgagagttgc | gccttcaggtttgacgccatt | 552 |
| aggrecan | [NM_007424.](http://www.ncbi.nlm.nih.gov/entrez/viewer.fcgi?db=nucleotide&val=6671522) | agagctatttccacacgctacac | gtaggttctcactccagggaact | 228 |
| Alkaline phosphatase | [X13409.](http://www.ncbi.nlm.nih.gov/entrez/viewer.fcgi?db=nucleotide&val=22138739) | acaccttgactgtggttactgct | ggaatgtagttctgctcatggac | 334 |
| α-MHC | [BC110700.](http://www.ncbi.nlm.nih.gov/entrez/viewer.fcgi?db=nucleotide&val=83405898) | gatggcacagaagatgctga | ctgccccttggtgacatact | 120 |
| aP2(Fabp4) | [NM_024406.](http://www.ncbi.nlm.nih.gov/entrez/viewer.fcgi?db=nucleotide&val=14149634) | ctggaagcttgtctccagtga | tatgatgctcttcaccttcctgt | 232 |
| BMP2 | [NM_007553](http://www.ncbi.nlm.nih.gov/entrez/viewer.fcgi?db=nucleotide&val=71896668) | tcttagacggactgcggtctc | cctgagtgcctgcggtacaga | 300 |
| α cardiac-actin | [BC062138.](http://www.ncbi.nlm.nih.gov/entrez/viewer.fcgi?db=nucleotide&val=38328336) | agcccagagcaagcgaggtat | agtcagtgaggtcccgaccag | 392 |
| Cardiac Troponin T | [NM_011619.](http://www.ncbi.nlm.nih.gov/entrez/viewer.fcgi?db=nucleotide&val=6755842) | gaggaggtggtggaggagta | ggcttcttcatcaggaccaa | 150 |
| Cbfa(Runx2) | [NM_009820.](http://www.ncbi.nlm.nih.gov/entrez/viewer.fcgi?db=nucleotide&val=70909357) | atccatccactccaccacgc | aagggtccactctggctttgg | 371 |
| CD34 | [BC006607.](http://www.ncbi.nlm.nih.gov/entrez/viewer.fcgi?db=nucleotide&val=13879273) | tatggaaaagcaccaatctgact | tctccgtgtaataagggtcttca | 221 |
| cdx2 | [NM_007673.](http://www.ncbi.nlm.nih.gov/entrez/viewer.fcgi?db=nucleotide&val=31560721) | gatacatcaccatcaggaggaaa | caaggaggtcacaggactcaag | 236 |
| c-fms | [NM_007779.](http://www.ncbi.nlm.nih.gov/entrez/viewer.fcgi?db=nucleotide&val=6681044) | taagcaagatctggacaaagagc | gagtcattcatgatgtccctagc | 207 |
| c-KIT | [NM_021099.](http://www.ncbi.nlm.nih.gov/entrez/viewer.fcgi?db=nucleotide&val=10947120) | aactccatgtggctaaagatgaa | ccagaaaggtataagtgcctcct | 460 |
| Collagen II | [NM_031163.](http://www.ncbi.nlm.nih.gov/entrez/viewer.fcgi?db=nucleotide&val=70980519) | aggggtaccaggttctccatc | ctgctcatcgccgcggtccta | 432/225 |
| Cripto-1 | [BC052646.](http://www.ncbi.nlm.nih.gov/entrez/viewer.fcgi?db=nucleotide&val=30851244) | tagcctttgggtgtttcgaga | cagtgctctttgcgaacatca | 395 |
| E-cad | [NM_009864.](http://www.ncbi.nlm.nih.gov/entrez/viewer.fcgi?db=nucleotide&val=6753373) | tatgatgaagaaggaggtggaga | aacaccaacagagagtcgtaagg | 236 |
| Eomesodermin | [NM_010136.](http://www.ncbi.nlm.nih.gov/entrez/viewer.fcgi?db=nucleotide&val=83921571) | agcttcaacataaacggactcaa | gaaggtctgagtcttggaaggtt | 366 |
| Flk-1 | [NM_010612.](http://www.ncbi.nlm.nih.gov/entrez/viewer.fcgi?db=nucleotide&val=27777647) | ctgaactcaagatcctcatccac | gatacacttccttgatgccaaga | 425 |
| Flt-1 | [NM_010228](http://www.ncbi.nlm.nih.gov/entrez/viewer.fcgi?db=nucleotide&val=34328179) | cagactcttgtcctcaactgcac | ggctctccacattgttgatctta | 175 |
| FSP-1 (S100a4), | NM_011311 | gtccaccttccacaaatactcag | ctcctggaagtcaacttcattgt | 184 |
| GAPDH | [BC085275.](http://www.ncbi.nlm.nih.gov/entrez/viewer.fcgi?db=nucleotide&val=55154586) | cagcaaggacactgagcaag | gggtgcagcgaactttattg | 156 |
| GATA4 | [NM_008092](http://www.ncbi.nlm.nih.gov/entrez/viewer.fcgi?db=nucleotide&val=46909568) | ggtgcctccagcggtaactcc | tgattacgcggtgattatgtc | 300 |
| GFAP | [NM_010277.](http://www.ncbi.nlm.nih.gov/entrez/viewer.fcgi?db=nucleotide&val=84000447) | ggatttggagagaaaggttgaat | tgcaaacttagaccgataccact | 234 |
| GLUT4 | [NM_009204.](http://www.ncbi.nlm.nih.gov/entrez/viewer.fcgi?db=nucleotide&val=6678014) | tccaactggacctgtaacttcat | caagttctgtactgggtttcacc | 226 |
| Hand1 | [NM_008213.](http://www.ncbi.nlm.nih.gov/entrez/viewer.fcgi?db=nucleotide&val=6680164) | atcatcaccatcatcaccactc | gcgccctttaatcctcttct | 581 |
| Ihh | [NM_010544.](http://www.ncbi.nlm.nih.gov/entrez/viewer.fcgi?db=nucleotide&val=31981670) | ggcctgaggttgtctccgtct | ggaaggtcatgtttcgggatg | 411 |
| LPL | [NM_008509](http://www.ncbi.nlm.nih.gov/entrez/viewer.fcgi?db=nucleotide&val=6678709) | agcagcaagatgtacctgaagac | ttctccctagcacagaagatgac | 374 |
| Mash2 (ASCL2) | [AF139595.](http://www.ncbi.nlm.nih.gov/entrez/viewer.fcgi?db=nucleotide&val=4928950) | agagtacattcggaccctctctc | ccaccttactcagcttcttgttg | 600 |
| Mef2c | [NM_025282](http://www.ncbi.nlm.nih.gov/entrez/viewer.fcgi?db=nucleotide&val=13384623) | agcactgacatggataaggtgtt | ggtgagtgcataagaggagtcag | 150 |
| Mesp1 | [NM_008588.](http://www.ncbi.nlm.nih.gov/entrez/viewer.fcgi?db=nucleotide&val=33469090) | agaacctgaccaagatcgagac | tctagaagagccagcatgtcg | 361 |
| Mesp2 | [NM_008589](http://www.ncbi.nlm.nih.gov/entrez/viewer.fcgi?db=nucleotide&val=6678863) | ctaggaacaagactggacactgg | aaagttcaggacagccactgag | 303 |
| MyoD | [NM_010866.](http://www.ncbi.nlm.nih.gov/entrez/viewer.fcgi?db=nucleotide&val=6996931) | ctacccaaggtggagatcctg | gtggagatgcgctccactat | 350 |
| Myogenin | [NM_031189](http://www.ncbi.nlm.nih.gov/entrez/viewer.fcgi?db=nucleotide&val=13654246) | tgtgtaagaggaagtctgtgtcg | agcaaatgatctcctgggttg | 349 |
| Myostatin | [AY204900.](http://www.ncbi.nlm.nih.gov/entrez/viewer.fcgi?db=nucleotide&val=28797966) | agctcctaacatcagcaaagatg | ggtttgatgagtctcaggatttg | 333 |
| Nanog | [XM_132755.](http://www.ncbi.nlm.nih.gov/entrez/viewer.fcgi?db=nucleotide&val=83029641) | gtggttgaagactagcaatggtc | tggtggagtcacagagtagttca | 433 |
| Nestin | [BC060693.](http://www.ncbi.nlm.nih.gov/entrez/viewer.fcgi?db=nucleotide&val=38173717) | gaggaagaagatgctgatgaaga | gccactgatatcaaaggtgtctc | 353 |
| NF-H | [NM_010904.](http://www.ncbi.nlm.nih.gov/entrez/viewer.fcgi?db=nucleotide&val=46391088) | gcccaagaggagataactga | ttctgtcactccttccgtcac | 477 |
| NF-M | [NM_008691.](http://www.ncbi.nlm.nih.gov/entrez/viewer.fcgi?db=nucleotide&val=6679047) | tagagcgcaaagattacctgaag | ttgacgttaaggagatcctggta | 406 |
| NKx.2.5 | [NM_008700.](http://www.ncbi.nlm.nih.gov/entrez/viewer.fcgi?db=nucleotide&val=76677926) | ccaaagaccctcgggcggata | gcgcagctgtagccgggactg | 493 |
| Nodal | [NM_013611](http://www.ncbi.nlm.nih.gov/entrez/viewer.fcgi?db=nucleotide&val=62996442) | ggcgcaagatgtggacgtgac | acattgtgctggcgacaggt | 402 |
| Oct4 | [M34381.](http://www.ncbi.nlm.nih.gov/entrez/viewer.fcgi?db=nucleotide&val=200117) | ggcgttctctttggaaaggtgttc | caaagctccaggttctcttg | 635 |
| Osteocalcin | [L24431,](http://www.jci.org/cgi/external_ref?access_num=L24431&link_type=GEN) | atgctactggacgctggagggt | gcggtcttcaagccatactggtc | 330 |
| Osteopontin | [NM_009263.](http://www.ncbi.nlm.nih.gov/entrez/viewer.fcgi?db=nucleotide&val=6678112) | atgaatctgacgaatctcaccat | cttagactcaccgctcttcatgt | 233 |
| p75NTR | [BC038365.](http://www.ncbi.nlm.nih.gov/entrez/viewer.fcgi?db=nucleotide&val=23468246) | attgctttcaagagatggaacag | ctactgtagaggttgccatcacc | 206 |
| pax-7 | [NM_011039](http://www.ncbi.nlm.nih.gov/entrez/viewer.fcgi?db=nucleotide&val=34328054) | agaaagaagaagatggcgagaag | gtagagtccggcagctggtag | 403 |
| Pecam1 | [AF412277S4](http://www.ncbi.nlm.nih.gov/entrez/query.fcgi?cmd=Retrieve&db=Nucleotide&list_uids=22595861&dopt=GenBank) | cacatctgcaatctcgggtgt | gaagaggcgattgtcacgttc | 268 |
| PPAR-γ | [NM_011146](http://www.ncbi.nlm.nih.gov/entrez/viewer.fcgi?db=nucleotide&val=6755137) | agaaccttctaactccctcatgg | cggcagttaagatcacacctatc | 159 |
| pref-1(DLK1) | [L12721.](http://www.ncbi.nlm.nih.gov/entrez/viewer.fcgi?db=nucleotide&val=309092) | gaaattctgcgaaatagacgttc | actcttgttgagctctttcatgg | 646 |
| Runx1 | [NM_009821](http://www.ncbi.nlm.nih.gov/entrez/viewer.fcgi?db=nucleotide&val=6753297) | tacctgggatccatcacctc | gacggcagagtagggaactg | 164 |
| Shh | [NM_009170](http://www.ncbi.nlm.nih.gov/entrez/viewer.fcgi?db=nucleotide&val=40254618) | ggaaggtgaggaagtcgctgt | ttggccatctctgtgatgaac | 372 |
| Smooth muscle α actin | [NM_007392.](http://www.ncbi.nlm.nih.gov/entrez/viewer.fcgi?db=nucleotide&val=31982518) | ctattcaggctgtgctgtcc | ccaagtccagacgatgat | 107 |
| Sox9 | [NM_011448](http://www.ncbi.nlm.nih.gov/entrez/viewer.fcgi?db=nucleotide&val=31543760) | cacggaacagactcacatctctc | aaggtctcaatgttggagatgac | 291 |
| Tbrachyury | NM_009309 | gctgttgggtagggagtcaag | aaattgggcgagtctgg | 446 |
| Tyrp1 | [NM_031202](http://www.ncbi.nlm.nih.gov/entrez/viewer.fcgi?db=nucleotide&val=50233911) | tccagaagcaacttcgattctac | tgttgtattgcctgttatgtcca | 517 |
| Wnt1 | [NM_021279.](http://www.ncbi.nlm.nih.gov/entrez/viewer.fcgi?db=nucleotide&val=47271537) | gccgagaaacagcgttcatc | ggttcatgaggaagcgtagg | 236 |
